# Design and experiment of transfer and loading machine for tobacco poles in bulk curing barn scenario

**DOI:** 10.3389/fpls.2026.1801854

**Published:** 2026-04-24

**Authors:** Ting Guo, Huyang Tang, Minghao Li, Cheng Liu, Chao Chen, Ling Wang

**Affiliations:** 1 Hunan Tobacco Company, Chenzhou, Hunan, China; 2 College of Engineering, China Agricultural University, Beijing, China

**Keywords:** finite element analysis (FEA), intensive curing barn, multi-stage lifting, tobacco curing, tobacco pole transport and loading machine

## Abstract

Tobacco leaf curing is a critical stage in the tobacco processing chain, and its operational methods and equipment technology levels are key factors affecting leaf quality, operational efficiency, and overall economic benefits. However, current curing practices are still dominated by manual operations, which are generally characterized by high labor intensity, low efficiency, high labor costs, and insufficient operational safety. These limitations hinder the large-scale, standardized, and efficient development of the tobacco curing process. To address these challenges, this study designed and developed a tobacco pole transfer and loading machine specifically for dense curing barn environments, based on the structural characteristics of dense curing barns and the technological requirements of tobacco pole loading. The proposed machine adopts a multi-stage lifting system combining a five-stage mast, servo motors, and ball screws, enabling precise vertical lifting within a range of 1.3~3.6 m. A reciprocating tobacco pole carrying mechanism, driven by staggered linear modules, allows adjustable pole inclination within a range of 0~32°, effectively avoiding interference with curing barn walls. Finite element analysis of key components indicates that the strength of major load-bearing structures, including the powered chassis and lifting mast, meets safety requirements. The natural frequencies of mast stages 1 to 6 range from 63.92 to 354.00 Hz, avoiding resonance during operation. In addition, the safety factor of the tobacco pole carrying blade is significantly higher than the allowable value, satisfying fatigue life design criteria. Field experiments conducted in tobacco-producing areas demonstrate that the loading success rate of the developed machine exceeds 90%, and operational efficiency is improved by approximately 3 to 5 times compared with traditional manual methods. The results confirm that the proposed tobacco pole transfer and loading machine can effectively accomplish pole transportation and hanging tasks in dense curing barns, substantially improving curing operation efficiency.

## 1 Introduction

Tobacco is an economically important crop cultivated extensively in China and worldwide, with its production and processing systems playing a significant role in agricultural production and related industries across many countries and regions (Zhang et al., 2008). Tobacco leaf curing represents a critical stage in post-harvest handling and primary processing, exerting a decisive influence on the development of physicochemical properties and the final processing performance of cured leaves (Li, 2018; Tie et al., 2009). With the ongoing transition of tobacco production toward large-scale, intensive, and specialized systems, dense curing barns have been widely adopted due to their high loading capacity, superior operational efficiency, and stable performance (Zhan et al., 2011). However, under dense curing barn conditions, the transportation and loading of fresh tobacco leaves are still predominantly reliant on manual labor. This practice is commonly associated with high labor intensity, low operational efficiency, elevated labor costs, and inadequate occupational safety, rendering it insufficient to meet the modern demands for efficient, standardized, and mechanized curing operations and thereby constraining further advancement of the tobacco leaf curing process (Li et al., 2024; Wang et al., 2021; Wu, 2021; Wu et al., 2020).

Since the 1960s, systematic research has been conducted on mechanized equipment for tobacco leaf loading and transportation. Most of the existing loading systems are based on loose-leaf curing modes, in which tobacco leaves are boxed during harvesting, thereby eliminating the need for stringing or bundling operations during curing. In such systems, leaf loading into curing barns is typically accomplished using forklifts or integrated lifting devices installed within the barns, resulting in a relatively high level of mechanization (Mote, 1899; Yousung, 2007; Haruzono, 1995). However, owing to substantial differences in barn structural configurations and loading procedures, the applicability of these loading systems remains limited under dense curing barn conditions.

In recent years, research addressing loading operations under dense curing barn conditions has predominantly focused on lifting-type loading structures, supplemented by movable curing rack systems. Development efforts have mainly emphasized improving operational efficiency while minimizing leaf damage. A tilt-gripping loading machine for dense curing barns was jointly developed by the Bijie Branch of Guizhou Tobacco Company and Southwest University (Fu et al., 2024). This system employs hydraulic actuation combined with a two-stage chain lifting mechanism to achieve vertical elevation of tobacco poles, while a push–linkage mechanism enables controlled pole tilting to facilitate smooth entry into the curing barn. Upon reaching the target loading position, the gripping mechanism releases the pole to complete the loading operation. The device can carry three tobacco poles per cycle, achieving more than a twofold increase in efficiency compared with manual operation; however, the use of a hydraulic system results in relatively high overall mass, thereby limiting mobility. Northwest A&F University developed a rotary tilting loading machine (Li et al., 2025), in which an electric push pole drives a rack-and-rotary-bearing mechanism to tilt tobacco poles by 15-20°, while a hydraulic-cylinder-driven scissor lift provides vertical elevation. The maximum lifting height of this system reaches 2.8 m, with a single-cycle capacity of four tobacco poles and a loading efficiency of up to 120 poles per hour. Nevertheless, the relatively large overall dimensions particularly the width-restrict maneuverability in narrow aisles within dense curing barns. The Tobacco Research Institute of the Chinese Academy of Agricultural Sciences developed a mobile hanging-type loading rack (Xiong et al., 2021), consisting mainly of a hanging rack, rails, and a hydraulic overturning platform. After the rack is laid flat, tobacco leaves are manually loaded; the rack is arranged in three layers, each equipped with two comb-type holders to secure the leaves. Once fully loaded, the rack is uprighted via the overturning platform and pushed into the curing barn for operation. South China Agricultural University designed a chain-lifting loading machine (Xie et al., 2021), in which tobacco pole conveyance is achieved through the rotation of left and right chains mounted on the frame. The chains are arranged with a longitudinal offset to maintain a tilted posture during lifting. The frame is installed on a scissor-type lifting mechanism actuated by a hydraulic cylinder, while a pole-aligning unit mounted at the upper section reorients the tilted poles to an upright position, thereby completing the loading process.

To address the limitations of existing tobacco leaf transport and loading equipment in dense curing barns—such as excessive platform width, poor pass-ability and maneuverability, and insufficient adaptability to barn structures and operational processes—this study designed and developed a tobacco pole transport and loading machine tailored to dense curing barn environments. Focusing on spatial constraints and the requirements of multi-layer loading operations, the proposed system adopts a five-stage mast-type lifting mechanism to achieve stable and precise elevation of tobacco poles across multiple rack levels. In combination with a reciprocating pole-carrying unit, the machine enables active posture adjustment of the poles, allowing automatic tilting, accurate alignment, and efficient hanging within confined spaces. Finite element analyses of key load-bearing and motion components verified the structural strength and operational safety of the system. Field trials conducted in production areas further demonstrated that the developed machine can reliably perform tobacco pole transport and loading operations under dense curing barn conditions, substantially reducing manual labor intensity and improving operational efficiency. This work provides a feasible technical solution for advancing the mechanization and efficiency of loading operations in dense curing barns.

## 2 Overall structure and performance analysis

### 2.1 Operating environment and design requirements

**Figure 1:**
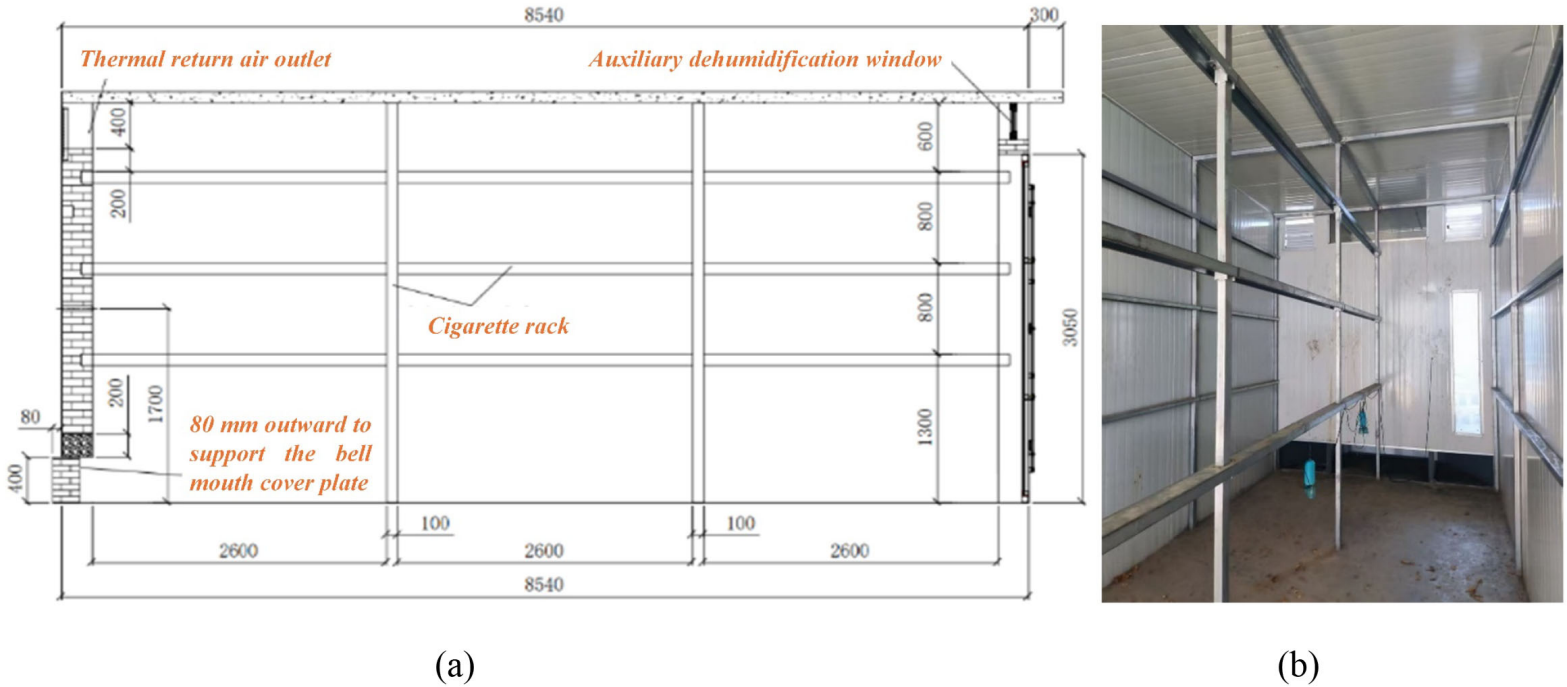
Schematic diagram of the intensive curing barn structure. **(a)** Sectional structural schematic of the loading chamber **(b)** Internal structure of the curing barn.

The dense curing barn used in this study was an updraft-type structure with an internal chamber measuring 8000 mm in length, 2700 mm in width, and 3500 mm in height. It is designed to accommodate more than 4500 kg of fresh tobacco leaves and to produce over 500 kg of cured leaves per batch. The barn mainly consists of tobacco hanging (loading) racks, airflow guide plates, a loading chamber door, and observation windows. The loading chamber is divided into left and right compartments, each equipped with bottom, middle, and top tiers of hanging (loading) racks. The heights of the bottom, middle, and top racks are 1300 mm, 2100 mm, and 2900 mm, respectively, with a clearance of 600 mm between the top rack and the roof. A schematic diagram of the dense curing barn structure is shown in **Figure 1a**.

**Figure 2:**
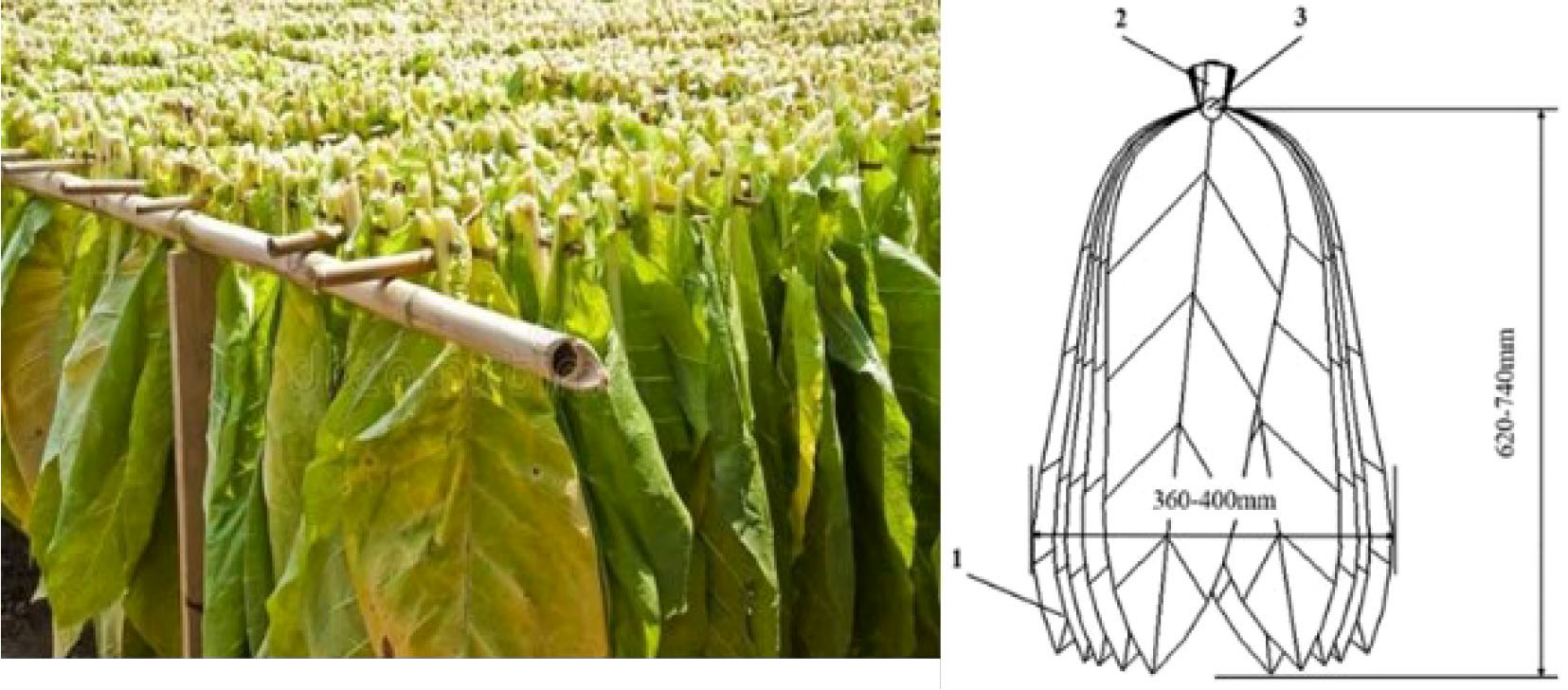
Schematic diagram of conventional tobacco stick loading. 1, Leaf tip; 2, Leaf base; 3, Conventional tobacco stick.

As shown in **Figure 1b**, the wooden tobacco poles are suspended at both ends on the hanging racks. The racks are fabricated from rectangular steel tubes with dimensions not less than 50 mm × 30 mm and a wall thickness of 3 mm, or from angle steel sections of 50 mm × 50 mm × 5 mm. The clear distance between the end faces of the hanging racks and the inner surfaces of the side walls is 1275 mm, and a doorstep approximately 50 mm in height is present at the entrance of the curing barn. The wooden tobacco poles used in this study have a length of 1360~1400 mm and a diameter of 27–33 mm. Each pole carries 43~48 bundles, with each bundle consisting of 2–3 leaves. Individual tobacco leaves are 750–820 mm in length and 269–330 mm in width. A clearance of 50–80 mm is reserved at each end of the pole to facilitate hanging. A typical loading configuration of a tobacco pole is illustrated in **Figure 2**. Measurements of fully loaded poles indicate an overall width of 360–400 mm, a distance of 620–740 mm from the pole to the leaf tips, and a total mass of 10–12 kg per pole. After loading the bottom tier, the distance from the leaf tips to the ground ranges from 550 to 590 mm. According to previous studies (Chen et al., 2014; Yang et al., 2017; Zhang, 2013), hanging density has a significant influence on tobacco leaf quality and curing efficiency. When the hanging density is maintained within the range of 60–65 kg/m³, the cured leaves exhibit higher contents of total sugar and reducing sugars, moderate levels of total nitrogen and nicotine, and lower contents of starch and chlorine, resulting in optimal curing performance. Under these conditions, approximately 421 tobacco poles are loaded in a single curing chamber, corresponding to a fresh leaf mass of 4200–4250 kg, with a spacing of 110–120 mm between adjacent poles.

To avoid interference between tobacco poles and the curing barn walls or hanging racks, and to ensure accurate lifting to the bottom, middle, and top tiers, the tobacco pole transport and loading machine is designed to meet specific functional requirements under dense curing barn conditions. The powered chassis is required to be compact, with overall dimensions smaller than 1200 mm in length, 800 mm in width, and 500 mm in height, while supporting in-place turning and basic obstacle-crossing capability to ensure maneuverability in confined spaces. The lifting system is designed as a multi-stage telescopic mechanism with a lifting range of 1.3-3.6 m to accommodate multi-layer hanging operations; meanwhile, the overall machine height in the non-lifting state was limited to 1.7 m to prevent structural interference within the barn. Given that the length of tobacco poles exceeds the width of individual curing chambers, the pole-carrying mechanism is required to provide horizontal inclination adjustment to enable smooth entry and lifting without collision, while maintaining leaf integrity and hanging accuracy. To improve operational efficiency, the carrying mechanism is designed to handle at least two poles per cycle, and a narrow-profile carrying blade is adopted to minimize mechanical damage to the leaves while satisfying structural strength requirements. In addition, the positioning accuracy of the machine is required to be within 10 mm to ensure reliable path tracking and stable, continuous operation during transport and hanging.

### 2.2 Structure and working principle

#### 2.2.1 Overall structural design

**Figure 3:**
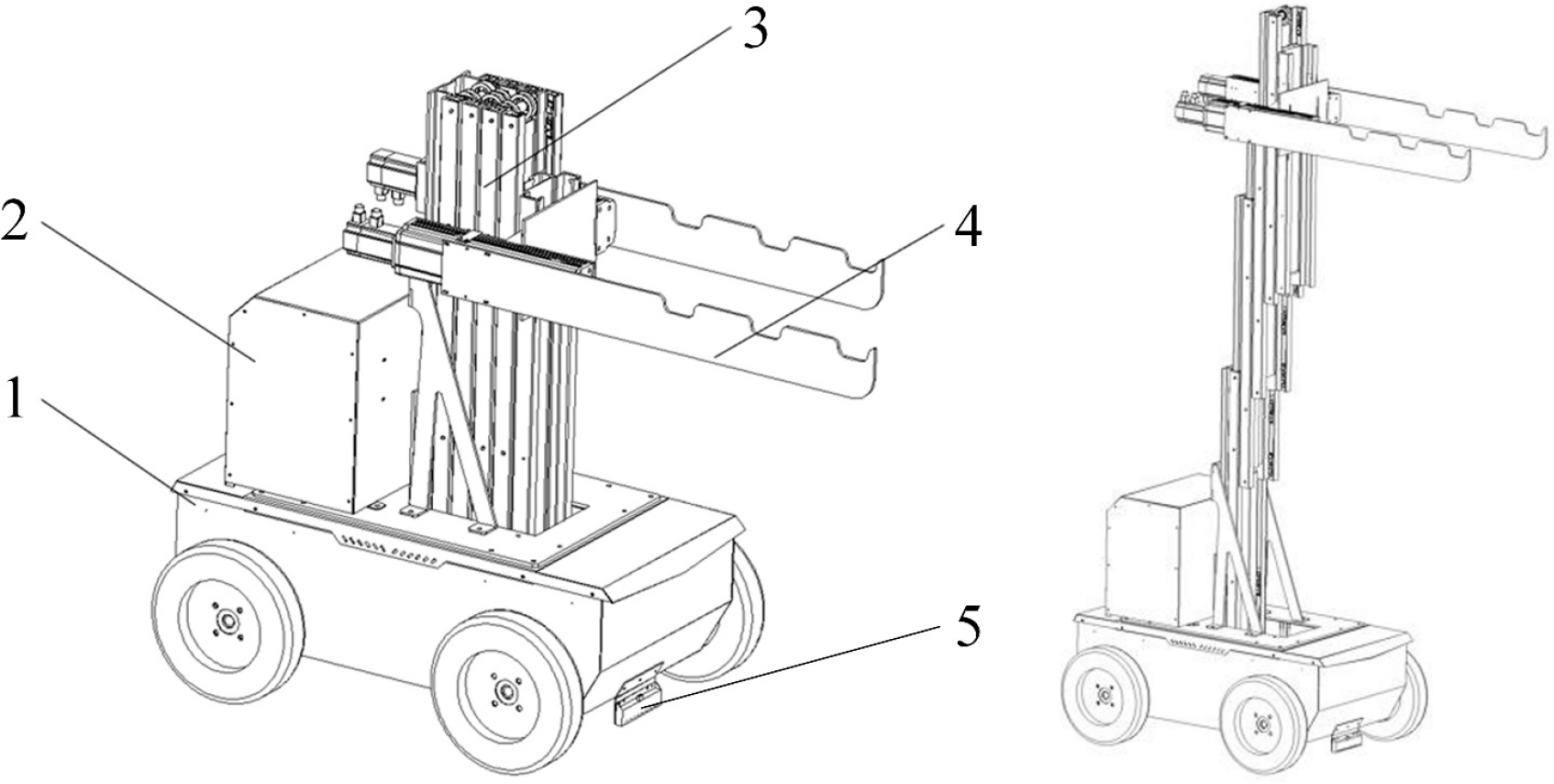
Schematic diagram of the tobacco stick transport and loading machine structure. 1, Power chassis; 2, Control box; 3, Lifting device; 4, Tobacco-carrying device; 5, Magnetic navigation sensor.

Based on the structural characteristics of dense curing barns and the tobacco pole hanging method, the tobacco pole transport and loading machine developed in this study mainly consists of a powered chassis, a control cabinet, a lifting system, and a pole-carrying mechanism. A schematic diagram of the machine structure is shown in **Figure 3**. The powered chassis is located at the base of the machine and houses the lifting system. Magnetic navigation sensors and Radio Frequency Identification (RFID) sensors are mounted at the front and lower sections of the chassis, respectively, to enable accurate identification and positioning along the transport path. Specifically, the magnetic navigation sensor is installed at the front underside of the chassis, close to ground level, to detect the magnetic strip laid on the floor for path tracking. The lifting system is installed in the central part of the powered chassis and primarily comprises an aluminum-alloy mast assembly and a servo motor-ball screw lifting mechanism, providing a maximum lifting height of 3.6 m. The pole-carrying mechanism is mounted at the front of the machine and connected to the final-stage mast via a mounting plate. A lightweight structural design is adopted to reduce the load on the front end of the system. The pole-carrying mechanism mainly consists of two linear motion modules and a carrying blade; horizontal inclination of the tobacco poles is achieved through the relative motion of the linear modules, effectively avoiding interference between the poles and the curing barn walls or hanging racks and thereby reducing mechanical damage to the tobacco leaves. The control cabinet is integrated at the rear of the machine to counterbalance the front-end load and contains key electrical components, including a 48 V/100 Ah lithium iron phosphate battery pack, a 220 V power inverter, and an embedded controller. After the predefined route and target hanging positions are set, the machine autonomously executes path tracking and hanging operations, while the loading of tobacco poles onto the carrying mechanism is performed manually by the operator, thus operating in a semi-automatic mode.

#### 2.2.2 Working principle

**Figure 4:**
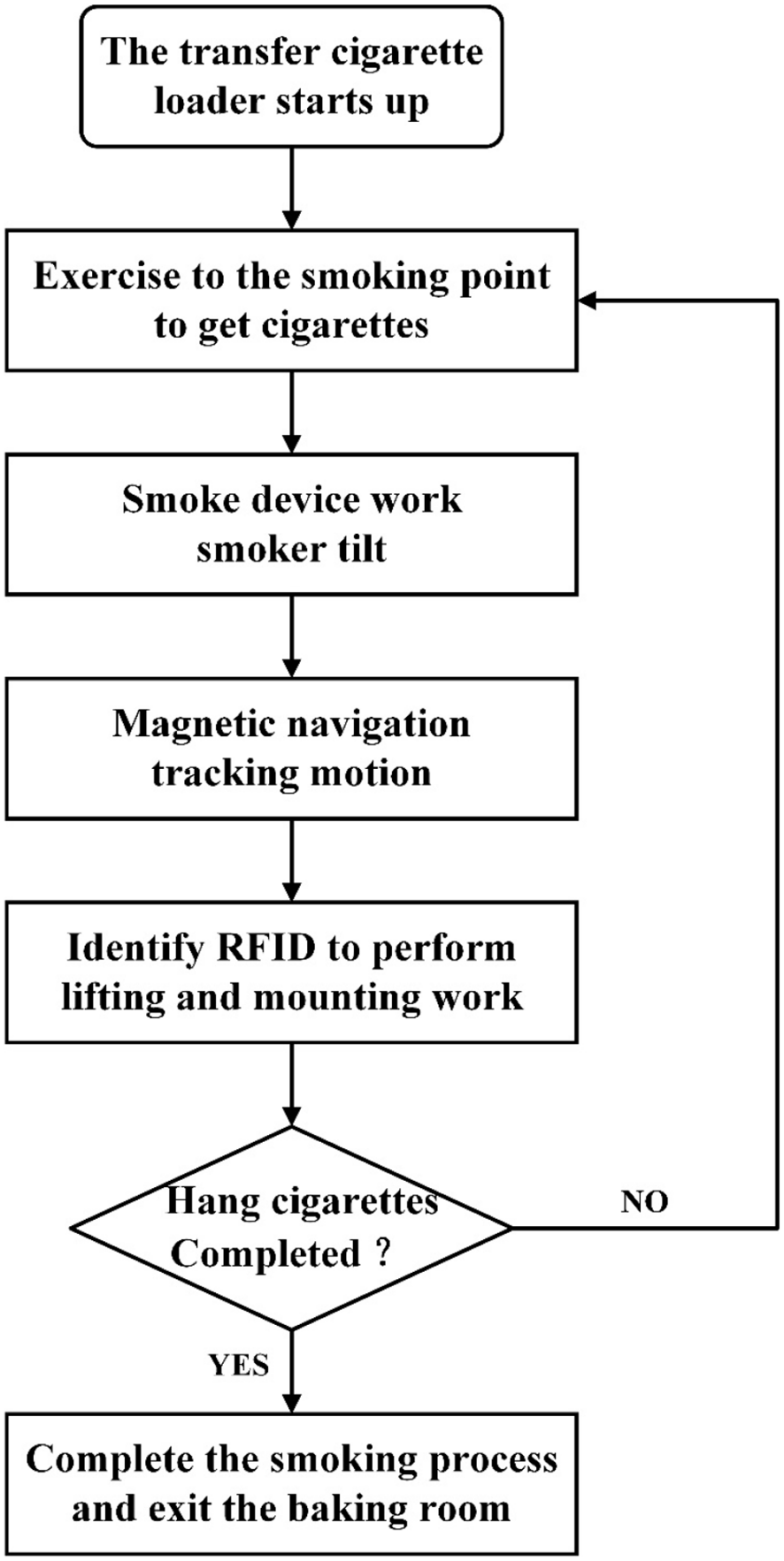
Operational flowchart of the tobacco stick transport and loading machine.

After start-up, the tobacco pole transport and loading machine moves to the pole pickup position, where operators place the prepared tobacco poles into the slots of the pole-carrying mechanism. Subsequently, the two linear motion modules within the carrying mechanism move synchronously in opposite directions. When the modules reach the limit switches, the motion automatically stops, completing the pole inclination process. Once the inclination is achieved, the navigation system is activated. The magnetic navigation sensors detect the magnetic strip to ensure that the machine follows the predefined path with high positional accuracy. During operation inside the curing barn, when the RFID sensor detects and reads a circular tag installed at a designated hanging position, the machine automatically stops and executes the corresponding hanging operation according to the tag information. Taking the hanging operation at the top tier as an example, after the RFID sensor identifies the target tag, the lifting system raises the tobacco poles to a height of 3.0 m above the ground. The two linear motion modules then operate again, returning the pole-carrying mechanism to its initial position and restoring the poles to a horizontal orientation. Subsequently, the lifting system descends, allowing the poles to settle onto the hanging rack under gravity, thereby completing the top-tier hanging process. The hanging procedures for the middle and bottom tiers follow the same sequence, differing only in lifting height and hanging position. After completing the hanging operation, to prevent mechanical damage to the tobacco leaves, the machine reverses along the magnetic navigation path to the curing barn entrance, performs an in-place turn, and then proceeds to the pole pickup position. At this point, one complete cycle of tobacco pole transport and hanging is accomplished. The overall operating procedure is illustrated in **Figure 4**.

#### 2.2.3 Performance analysis

(1) Anti-tipping stability analysis under critical operating conditions

**Figure 5:**
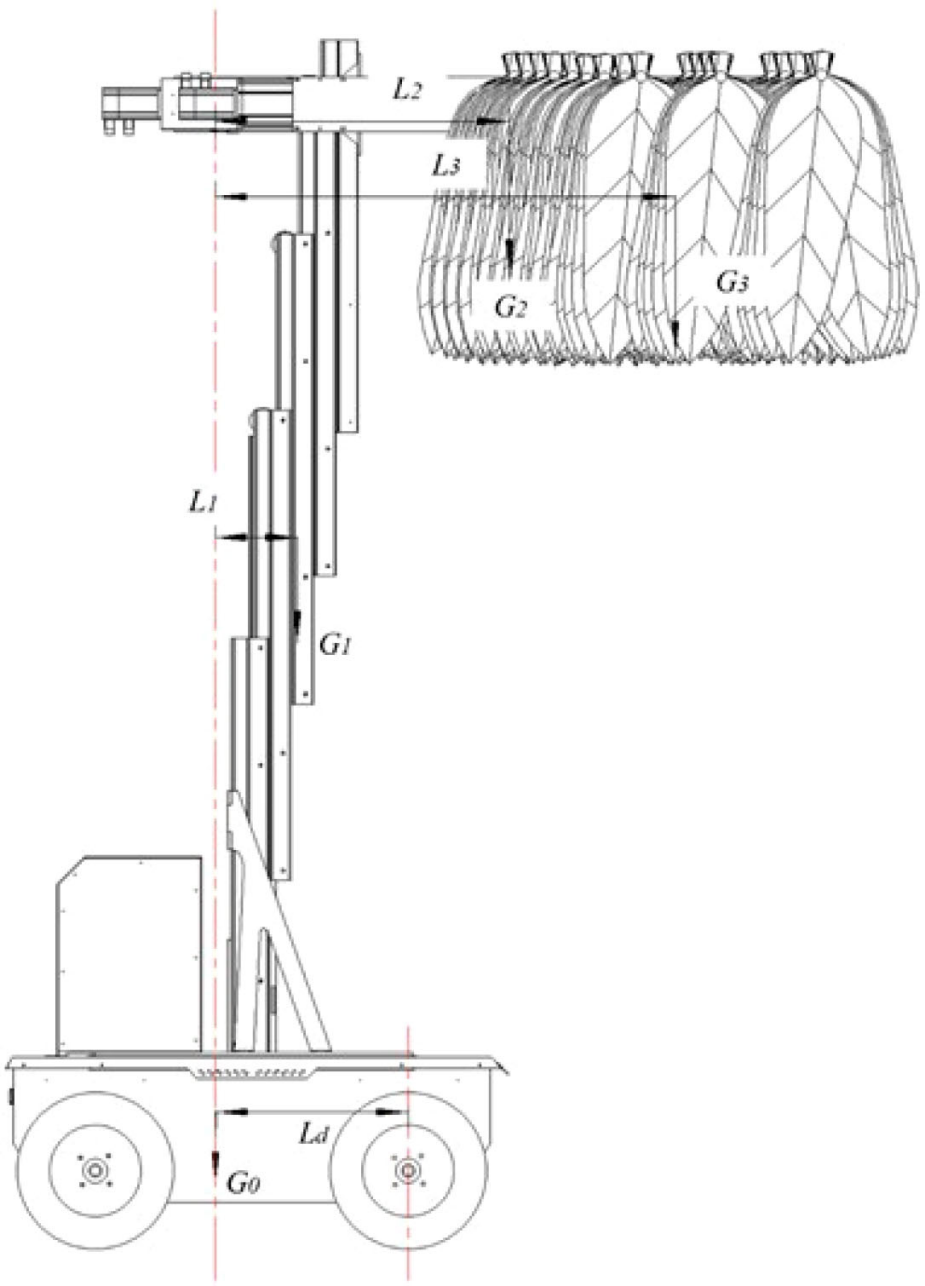
Schematic diagram of the machine under the critical operating condition at maximum extension.

Anti-overturning stability is a critical performance indicator for ensuring stable operation of the transport and loading machine and preventing tipping during operation (Zhang, 2013). According to the operating procedure of the machine, the center of gravity reaches its highest position when the machine is fully loaded with tobacco poles and lifted to the maximum height. Under this condition, the overturning moment is maximized, representing the most critical operating scenario (as shown in **Figure 5**). The position of the overall center of gravity forms the basis for evaluating anti-overturning stability under this extreme condition. Accordingly, material properties were assigned to all components in SolidWorks, and the mass properties evaluation tool was used to calculate the combined center of gravity of the entire machine under the critical operating condition. The results indicate that the composite center of gravity is located 490 mm from the center of the front wheels, corresponding to a center-of-gravity offset distance of *L_d_* = 490 mm.

Taking the center point of the front wheels as the pivot for analysis, moments are calculated for $G_{0}$, $G_{1}$, $G_{2}$ and $G_{3}$, respectively. Since $L_{2}$ and $L_{3}$ are greater than $L_{d}$, $G_{0}$ and $G_{1}$ produce stabilizing moments, while $G_{2}$ and $G_{3}$ produce overturning moments. The overturning moment is calculated according to Equation 1, and the stabilizing moment is calculated according to Equation 2.

(1) $$M_{T}=G_{2}\left(L_{2}-L_{d}\right)+G_{3}\left(L_{3}-L_{d}\right)$$

(2) $$M_{s}=G_{0}L_{d}+G_{1}\left(L_{d}-L_{1}\right)$$

Where,

*M_T_* is the overturning moment, *N·m*;

$G_{2}$ is the total mass of the tobacco-carrying device, *kg*;

$L_{2}$ is the horizontal distance between the center of gravity of the pole-carrying mechanism and the center of gravity of the powered chassis, *m*;

$L_{d}$ is the horizontal distance between the center of gravity of the powered chassis and the center point of the front wheels, *m*;

$G_{3}$ is the total mass of the tobacco sticks, *kg*;

$L_{3}$ is the horizontal distance from the CoG of the tobacco sticks to the CoG of the power chassis, *m*;

$M_{s}$ is the stabilizing moment, *N·m*;

$G_{0}$ is the total mass of the power chassis, *kg*;

$G_{1}$ is the total mass of the lifting device, *kg*;

$L_{1}$ is the horizontal distance from the Center of Gravity(CoG) of the lifting device to the CoG of the power chassis, *m*.

The main technical specifications of the tobacco stick transport and loading machine are shown in **Table 1**. Through measurement, some of the main parameters of the transport and loading machine are shown in **Table 2**.

**Table 1:** Technical specifications of the tobacco stick transport and loading machine.

Items	Units	Parameters
Maximum dimensions of the machine	mm	1780×850×1560
Total mass of the machine	kg	423
Travel speed	m/s	0.7
Loading capacity	kg	45~55
Maximum lifting stroke	mm	3600
Lifting speed	m/s	0.5
Tilting speed	m/s	0.3
Battery voltage	V	48

**Table 2:** Key physical and geometric parameters of the transport and loading machine.

Parameter/(units)	Values
*G_0_*	337
*G_1_*	83
*G_2_*	45
*G_3_*	40
*L_d_*	0.49
*L_1_*	0.21
*L_2_*	0.75
*L_3_*	1.17

By combining Equation 1 and Equation 2, Equation 3 is obtained as follows:

(3) $$K_{w}=\frac{M_{S}}{M_{T}}=\frac{G_{0}L_{d}+G_{1}\left(L_{d}-L_{1}\right)}{G_{2}\left(L_{2}-L_{d}\right)+G_{3}\left(L_{3}-L_{d}\right)}$$

By substituting the data from **Table 2**–2 into the equations, the anti-tipping stability safety factor (*K_w_*) of the transport and loading machine is calculated to be 4.84. This value is significantly greater than the safety threshold of 1.5, which is consistent with commonly adopted design safety factor principles for mechanical systems (Budynas and Nisbett, 2015), demonstrating that the machine poses no risk of overturning under normal operating conditions.

(2) Longitudinal stability analysis

**Figure 6:**
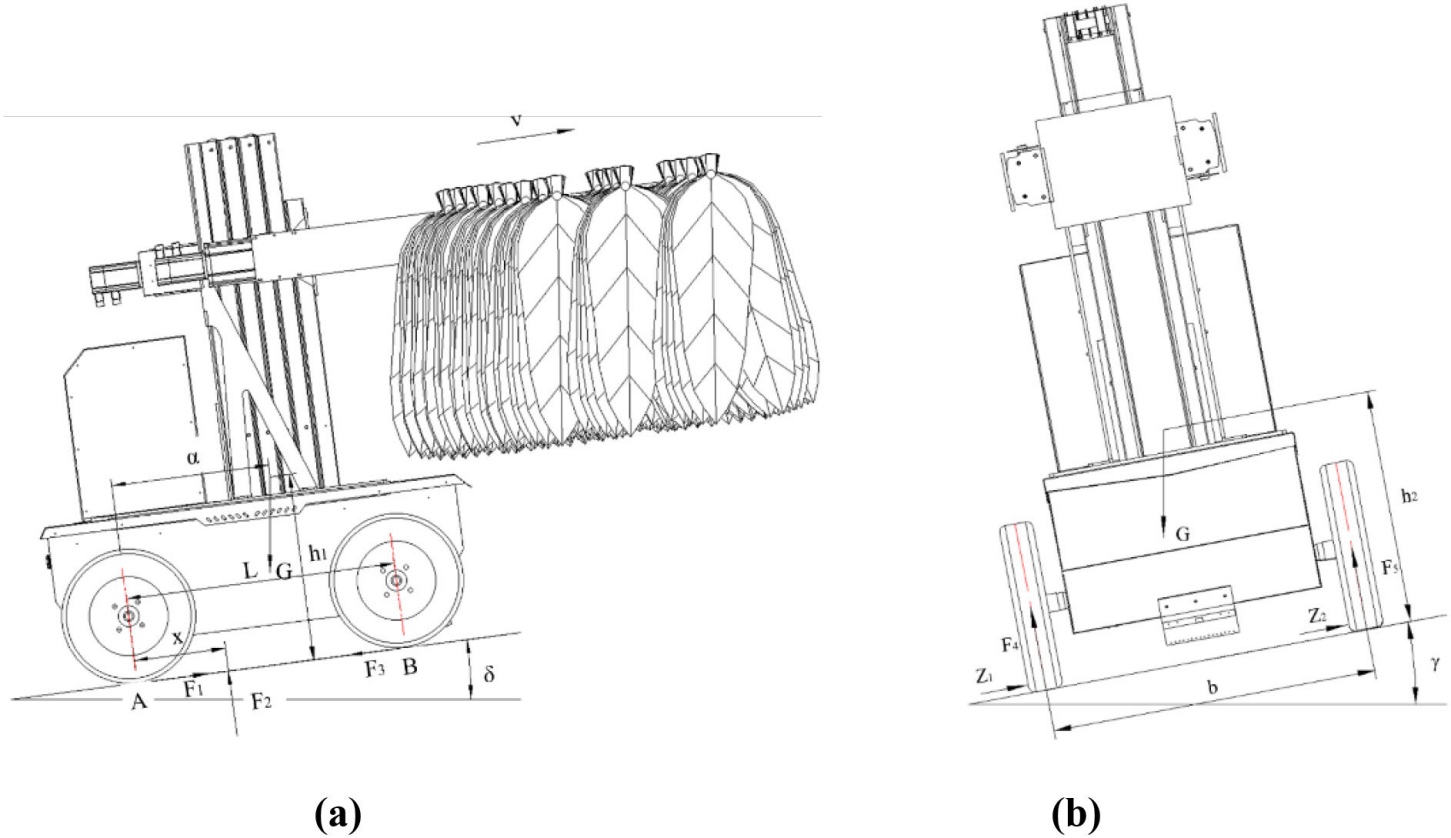
Force diagram of the transport and loading machine on an inclined plane. **(a)** Force diagram for uphill climbing **(b)** Force diagram for side-slope operation.

Longitudinal stability refers to the machine’s capability to resist overturning or sliding while operating on a slope of a certain angle. Given the specific operating environment of intensive curing barns, this study analyzes the longitudinal stability by focusing on the tilting condition as the transport and loading machine crosses the barn threshold. Considering that the maximum operating speed of the designed machine is only 4.0km/h (a low-speed operating condition), the effects of aerodynamic forces and inertial loads can be neglected. On this basis, a static stability analysis method is employed to evaluate the machine’s longitudinal stability (Yang et al., 2017). **Figure 6** illustrates the force diagram of the machine during an uphill climb. Based on the moment balance, Equation 4 is obtained, from which Equation 5 can be further derived as follows:

(4) $$F_{2}x+Gh_{1}\sin\delta -G\alpha \cos\delta =0$$

(5) $$x=\frac{G\left(\alpha \cos\delta -h_{1}\sin\delta \right)}{F_{2}}$$

Where,

G is the gravity of the transport and loading machine, *N*;

$h_{1}$ is the distance from the CoG of the machine to the slope, *mm*; α is the distance from the CoG of the machine to the center of the rear wheels, *mm*;

$F_{2}$ is the normal support forces from the ground acting on the machine, *N*; δ is the longitudinal tipping angle of the power chassis,*°*.

Analysis of Equation 5 reveals that as the slope angle increases, the point of application of the normal support force shifts toward the contact point A between the rear wheels and the ground. When the normal support force coincides with point A the system reaches a critical equilibrium state, at which point the chassis faces a risk of instability and overturning. To ensure the stability of the chassis system, the following condition must be met: the point of application of the normal support force must consistently remain in the region in front of the rear wheel contact point A, thereby maintaining a stable moment balance relationship. That is, Equation 6:

(6) $$x=\frac{G\left(\alpha \cos\delta -h_{1}\sin\delta \right)}{F_{2}}\geq 0$$

(7) $$\delta \leq \arctan\left(\frac{\alpha }{h_{1}}\right)$$

By substituting the relevant parameters into Equation 7, the maximum tipping angle of the tobacco pole transport and loading machine during uphill operation was determined to be 40.3°. Furthermore, the maximum tipping angle is closely related to the horizontal distance α between the overall center of gravity and the center of the rear wheels, as well as the vertical distance $h_{1}$ between the center of gravity and the slope surface. An increase in α and a decrease in $h_{1}$ enhance the overall stability of the machine, thereby reducing the likelihood of overturning.

In the context of intensive curing barns, the transport and loading machine may encounter side-slope conditions during operation, causing the entire vehicle to be in a laterally tilted state. To analyze the mechanical characteristics of the machine under such conditions, a lateral slope force analysis model is established (Chen et al., 2014). The corresponding free-body diagram is illustrated in **Figure 6b**.

Where,

b is the track width of the power chassis, mm; $h_{2}$ is the distance from the center of gravity (CoG) of the machine to the lateral slope, mm;

γ is the lateral tipping angle of the power chassis, °;

$F_{4}$, $F_{5}$ are the normal ground reaction forces acting on the left and right wheels, respectively, N;

$Z_{1}$, $Z_{2}$ are the lateral slip resistance forces acting on the left and right wheels, respectively,N.

The formula for calculating the critical lateral tipping angle (γ*_max_*) of the transport and loading machine is given by Equation 8:

(8) $$\gamma_{\max}=\arctan\left(\frac{b}{2h_{2}}\right)=\arctan\left(\frac{772}{2\times 553}\right)={\mathrm{34.91}}^{\circ }$$

The critical lateral tipping angle of the transport and loading machine is calculated to be 34.91°. Based on field measurements of the actual operating conditions in intensive curing barns, the maximum lateral slope angle is approximately 20°, which is significantly smaller than the critical tipping angle. These results demonstrate that the lateral stability design of the machine fully satisfies the requirements of practical operating environments.

### 2.3 Design of key components

#### 2.3.1 Power chassis

**Figure 7:**
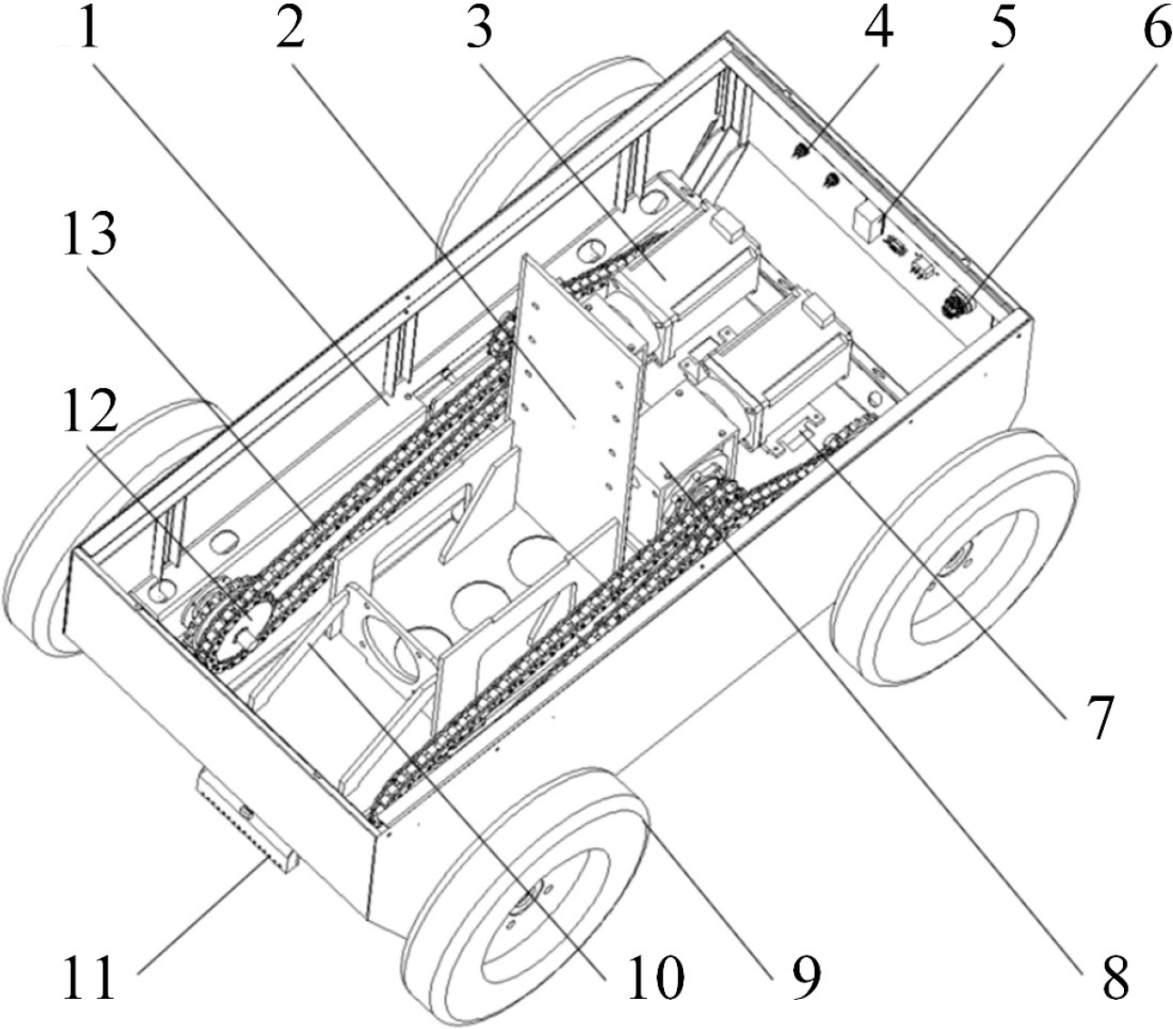
Structural schematic diagram of the power chassis. 1, Chassis Frame; 2, Lifting device mounting plate; 3, Drive motor; 4, Ultrasonic sensor; 5, Air circuit breaker; 6, Emergency stop button; 7, RFID sensor; 8, Worm gear reducer; 9, Rubber tire; 10, Servo motor mounting plate; 11, Magnetic navigation sensor; 12, Double-row sprocket; 13, Transmission chain.

As the core load-bearing and driving unit of the tobacco stick transport and loading machine, the power chassis primarily consists of the chassis frame, the propulsion system, and other functional components. The chassis frame includes the structural skeleton, base plate, and side panels. The propulsion system is composed of drive motors, worm gear reducers, double-row sprockets, and chains. The drive motor transmits power through the worm gear reducer to the double-row sprocket at the output end; subsequently, the chain drives the two tires on the same side to rotate at the same angular velocity. In addition to the frame and propulsion system, several functional components are integrated into the chassis: a magnetic navigation sensor is installed at the front of the base plate; the middle section houses the servo motor mounting plate, the lifting device mounting plate, and an RFID sensor; the rear section is equipped with an ultrasonic sensor, an air circuit breaker, and an emergency stop button to ensure operational safety and system emergency response capabilities. The specific structure is illustrated in **Figure 7**.

**Figure 8:**
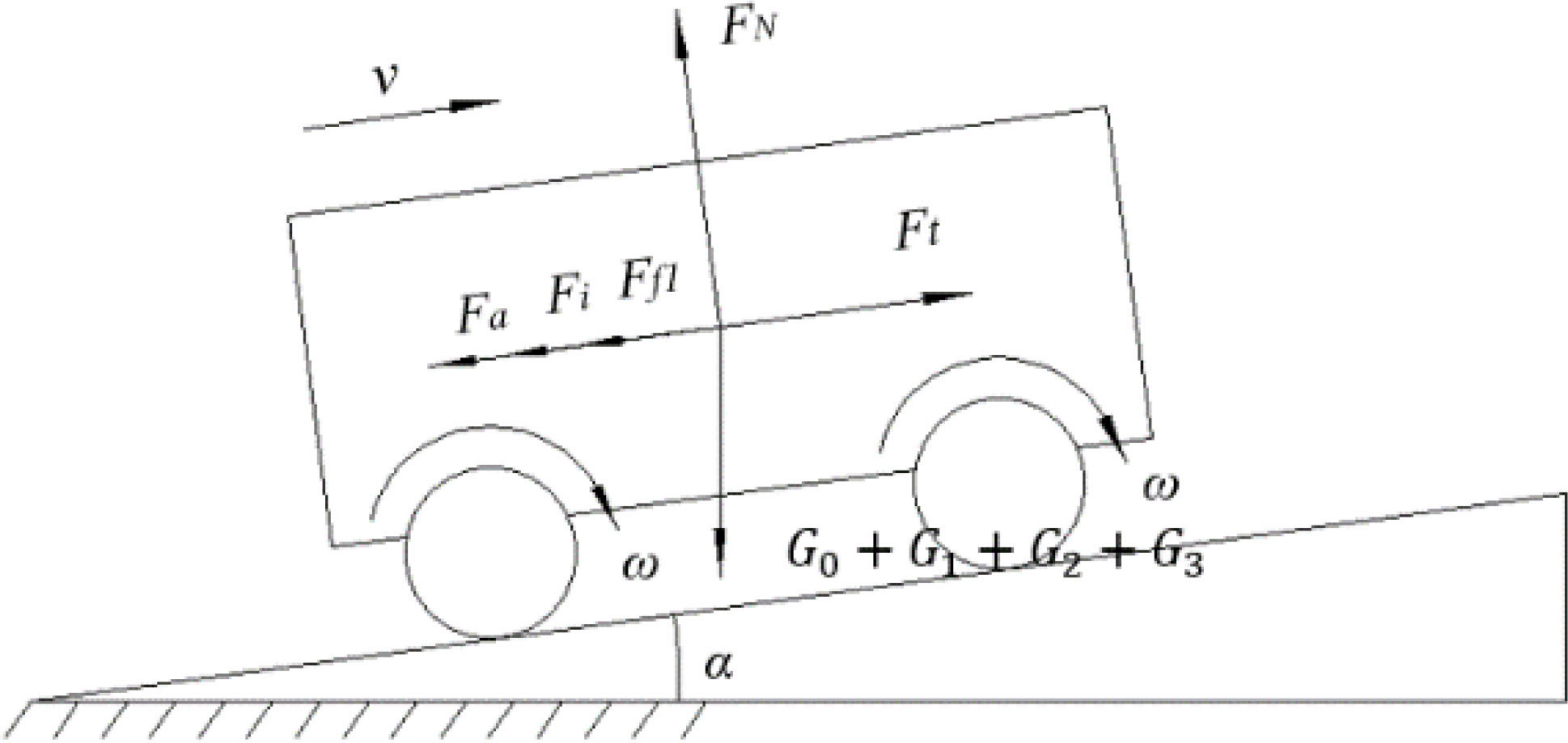
Force analysis diagram of the power chassis during climbing.

Given the uneven ground surfaces in typical operating environments, the climbing capability is a critical performance indicator for the transport and loading machine during tobacco stick transfer operations on inclined terrain. Neglecting the aerodynamic resistance during the climbing process, the forces acting on the power chassis are illustrated in **Figure 8**.

(9) $$F_{t}=F_{all}=F_{f1}+F_{i}+F_{a}$$

Where,

$F_{all}$ is the sum of resistances, *N*;

$F_{t}$ is the driving force of the power chassis, *N*;

$F_{f1}$ is the rolling resistance, *N*;

$F_{i}$ is the grade resistance, *N*; $F_{a}$ is the acceleration resistance, *N*.

The calculation formulas for each resistance component are given in Equation 10:

(10) $$\{\begin{array}{l} F_{f1}=\left(G_{0}+G_{1}+G_{2}+G_{3}\right)\cdot g\cdot f\cdot \cos\alpha \\ F_{i}=\left(G_{0}+G_{1}+G_{2}+G_{3}\right)\cdot g\cdot \sin\alpha \\ F_{a}=\left(G_{0}+G_{1}+G_{2}+G_{3}\right)\cdot a \end{array}$$

Where,

$G_{0}$ is the total mass of the power chassis,kg;

$G_{1}$ is the total mass of the lifting device, kg;

$G_{2}$ is the total mass of the tobacco-carrying device, kg;

$G_{3}$ is the total mass of the tobacco sticks, kg;

f is the rolling resistance coefficient, taken as 0.05;

α is the slope angle,°;

a is the gravitational acceleration, taken as9.8 m/s^2^.

Based on field investigations and measurement data, the terrain slope in the operating areas of local intensive curing barns primarily ranges from 10° to 12°. To ensure the reliability and adaptability of the transport and loading machine in complex environments, the operating condition for this study is set to a 20° slope. The machine’s traveling speed is defined as v=1m/s with an acceleration of a=1 m/s^2^. According to the Mechanical Design Handbook, the transmission efficiency of the worm gear is taken as $\lambda_{1}$=0.90, and the efficiency of the chain drive is taken as $\lambda_{2}$=0.98.

According to Equation 9, the required driving force ( $F_{t}$) for the power chassis is 2430.19 N. Based on Equation 11, the required output power for each motor is calculated to be 1406 W. Therefore, to ensure sufficient power redundancy, two drive motors with a rated output power of 1.5 kW each were selected for the power chassis.

(11) $$P=\frac{F_{t}\cdot v}{2\lambda }=1406W$$

#### 2.3.2 Lifting device

**Figure 9:**
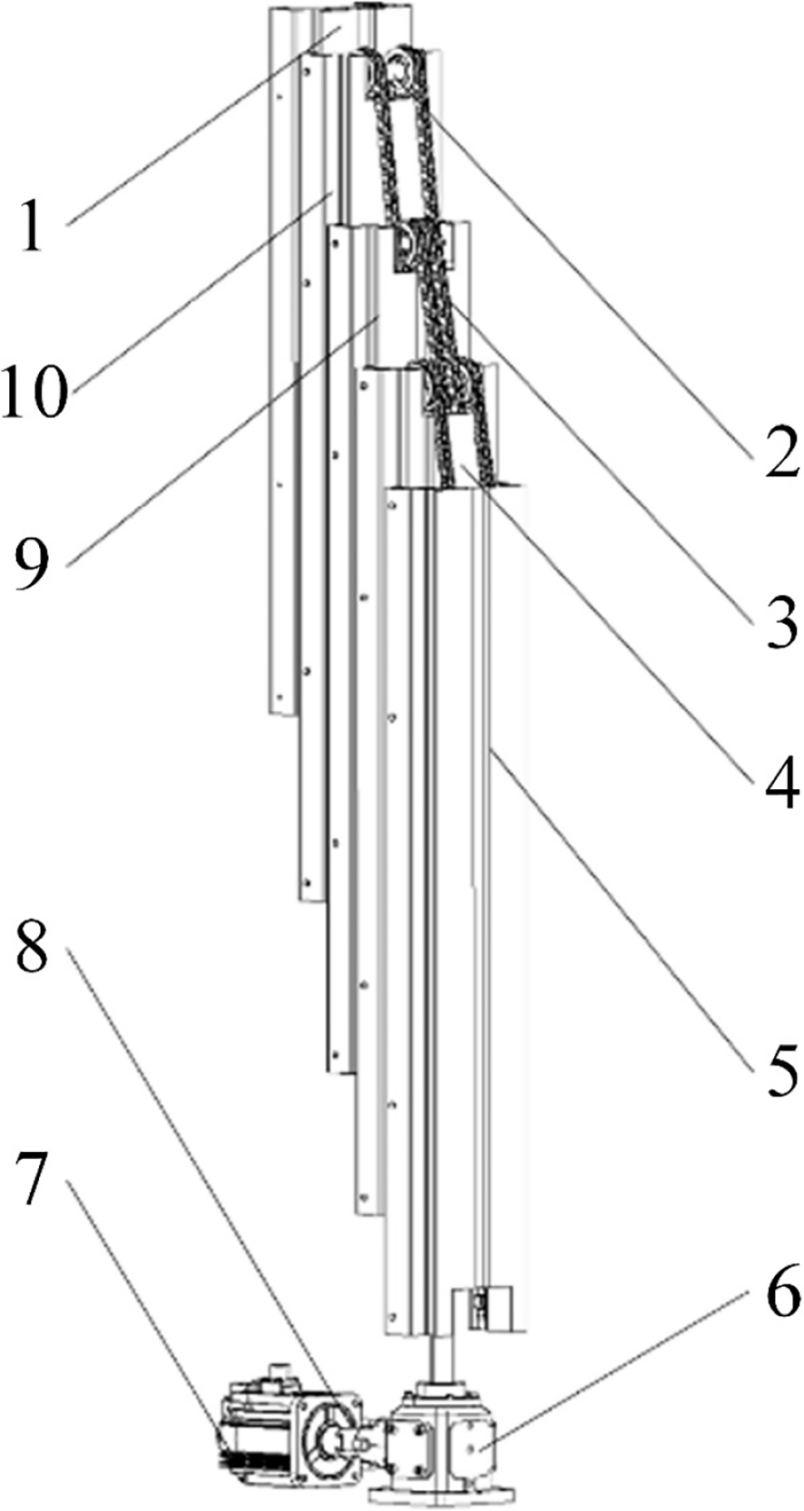
Structural schematic diagram of the multi-stage lifting device. 1, Fifth-stage mast; 2, Sprocket group; 3, Lifting chain; 4, Second-stage mast; 5, First-stage mast; 6, Screw elevator; 7, Servo motor; 8, Plum blossom coupling; 9, Third-stage mast; 10, Fourth-stage mast.

The multi-stage lifting system consists of a servo motor, a screw jack, and a five-stage mast assembly, as illustrated in **Figure 9**. The servo motor is mounted on the front section of the powered chassis via a mounting plate, and its output shaft is connected to the input shaft of the screw jack reducer through a jaw (plum-type) coupling. Inside the reducer, a pair of bevel gears with a reduction ratio of 2.5 is used to transmit torque and reduce rotational speed. A flange mounted on the screw jack is connected to the base of the second-stage mast, providing the driving force for the entire mast assembly. The mast assembly comprises five stages, each with a length of 1.2 m, resulting in a maximum lifting height of 3.6 m, which fully satisfies the hanging requirements for the bottom, middle, and top tiers. The first-stage mast is bolted to the lifting system mounting plate on the powered chassis. The second-, third-, and fourth-stage masts are each equipped with sprocket assemblies. Through the sprockets mounted on the second-stage mast, lifting chains connect the first-stage mast to the third-stage mast. The chains are fixed to chain clamps on the first- and third-stage masts. The sprocket assemblies and lifting chains on the remaining masts are installed in the same manner, thereby enabling synchronized motion of all mast stages.

**Figure 10:**
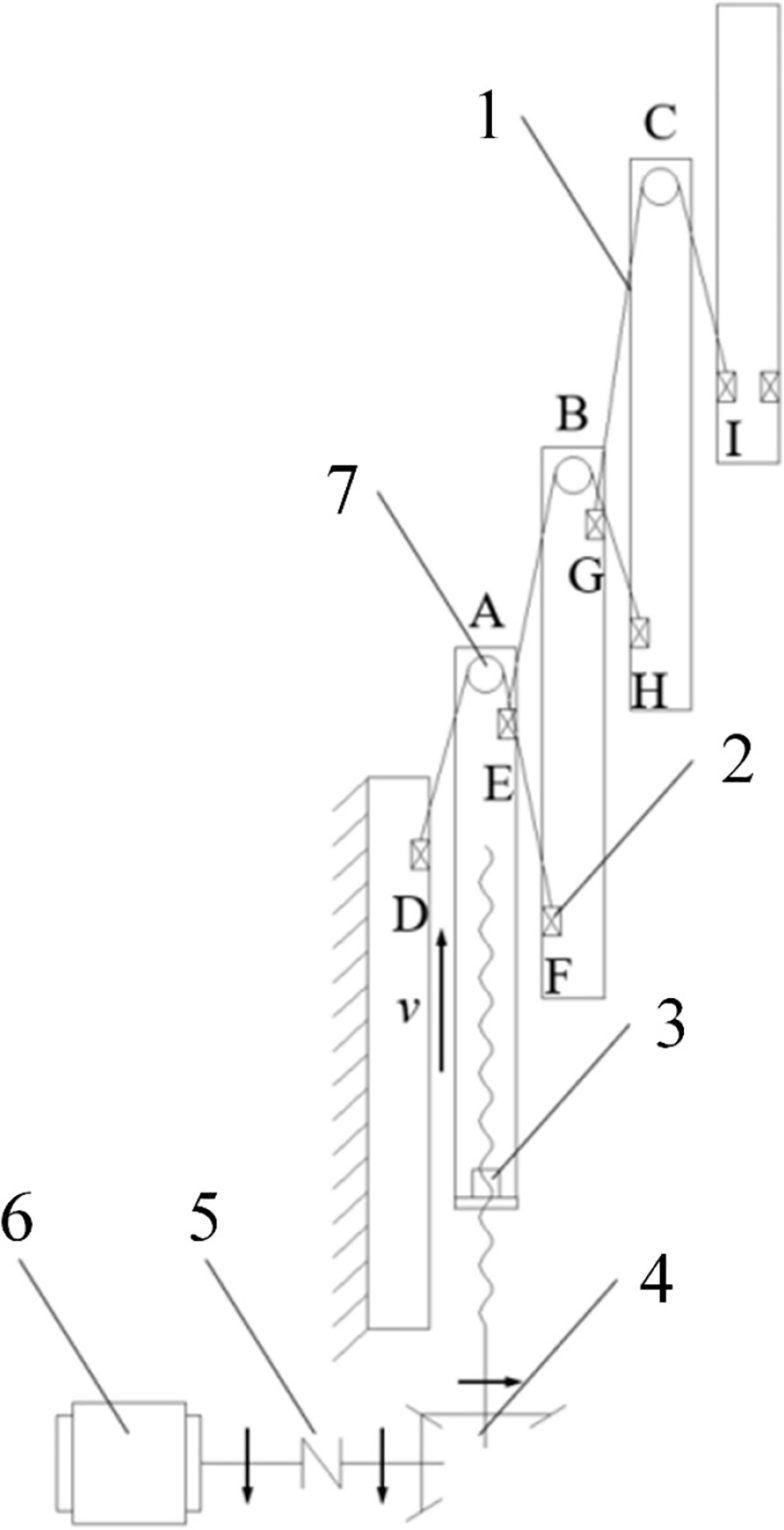
Transmission schematic of the multi-stage lifting device. 1, Lifting chain; 2, Chain clamp; 3, Flange seat; 4, Bevel gear reducer; 5, Coupling; 6, Motor power input; 7, Sprocket group.

When the multi-stage lifting device is in operation, the output shaft of the servo motor and the input shaft of the reducer rotate clockwise simultaneously via the coupling. Through the bevel gear transmission within the reducer, the lead screw shaft is driven to rotate counter-clockwise. This rotational motion is converted into the upward linear displacement of the flange seat, which pushes the second-stage mast upward. During this process, the length of chain segment AD increases while segment AF decreases. Since the total length of the chain is fixed and the first-stage mast is secured to the mounting plate, the third-stage mast is lifted under the traction of chain segment AF. Similarly, as the length of chain segment EH remains constant, an increase in the length of segment BE results in a corresponding decrease in segment BH thereby driving the fourth-stage mast upward. By following this mechanism, all stages are extended until the fifth-stage mast reaches the target height. Throughout the ascending process, the motion of all mast stages (except for the first stage) is synchronized. When the servo motor output shaft rotates counter-clockwise, the ball screw rotates clockwise, causing the flange seat to move vertically downward. The mast stages then return to their initial positions in the reverse order of the ascending process, aided by gravity. The transmission schematic of the multi-stage lifting device is shown in **Figure 10**.

**Figure 11:**
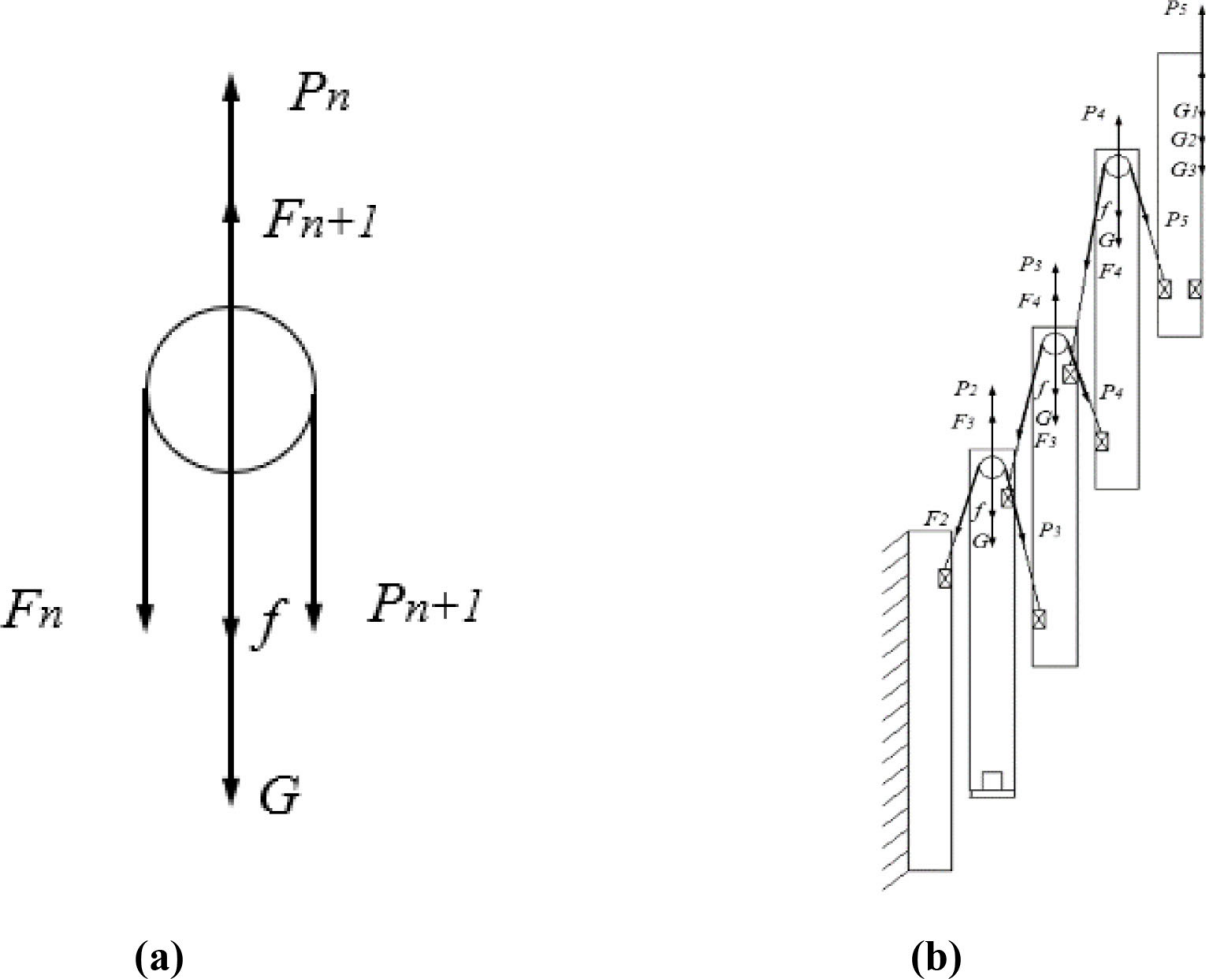
Schematic diagrams of force analysis for the sprockets and mast assembly. **(a)** Force analysis of the sprocket **(b)** Force analysis of the mast assembly.

According to the working principle of the multi-stage lifting device, the ball screw generates a vertical upward thrust to lift the second-stage mast. This power is subsequently transmitted to the following mast stages through components such as sprocket groups and lifting chains, enabling the upward motion of the entire assembly. The force analysis of the sprocket and chain on the $n^{th}$ stage mast is shown in **Figure 11(a)**. The primary forces acting on the sprocket include: Chain tension ( $F_{n}$): The force exerted by the chain on the sprocket, which also serves as the driving force required to lift the ${\left(n+1\right)}^{th}$ stage mast. Mast lifting force ( $P_{n}$): The force that drives the $n^{th}$ stage mast upward. For the second-stage mast, P_n_ is the thrust generated by the ball screw; for subsequent stages, P_n_ originates from the tension generated by the ${\left(n-1\right)}^{th}$ stage chain. Self-weight ( G): The gravitational force acting on an individual mast stage. Interaction resistance ( f): The frictional resistance generated by the interaction between two adjacent mast stages.

The overall force distribution of the mast assembly is illustrated in **Figure 11(b)**. From this, the system of force equilibrium Equation 12 can be established as follows:

(12) $$\{\begin{array}{c} {\text{F}}_{\text{n}}={\text{P}}_{\text{n}+1}\\ {\text{P}}_{5}=\text{k}\cdot \left({\text{G}}_{1}+{\text{G}}_{2}+{\text{G}}_{3}\right)\cdot g\\ {\text{P}}_{4}={\text{F}}_{4}+{\text{P}}_{5}+\text{f}+\text{k}\cdot \text{G}\cdot \text{g}\\ {\text{P}}_{3}={\text{F}}_{3}+{\text{P}}_{4}+\text{f}+\text{k}\cdot \text{G}\cdot \text{g}-{\text{F}}_{4}\\ {\text{P}}_{2}={\text{F}}_{2}+{\text{P}}_{3}+\text{f}+\text{k}\cdot \text{G}\cdot \text{g}-{\text{F}}_{3} \end{array}$$

Where,

k is the dynamic load coefficient of the chain;

$G_{1}$ is the mass of the fifth-stage mast, kg;

$G_{2}$ is the total mass of the tobacco-carrying device, kg;

$G_{3}$ is the total mass of the tobacco sticks, kg;

G is the gravitational acceleration, taken as9.8m/s2.

Based on the relevant data provided in **Table 2**, the force distribution for each mast stage is summarized in **Table 3**.

**Table 3:** Force analysis of each mast stage.

Parameters	Stage 5	Stage 4	Stage 3	Stage 2	Stage 1
Self-weight of the mast(kg)	7	11	11	11	11
Dynamic load coefficient of the chain	1.2	1.2	1.2	1.2	1.2
Inter-stage resistance(N)	8	8	8	8	8
Chain tension(N)	0	1081.92	2301.20	3657.84	5151.84
Mast lifting force(N)	1081.92	2301.20	3657.84	5151.84	

#### 2.3.3 Tobacco-carrying device

**Figure 12:**
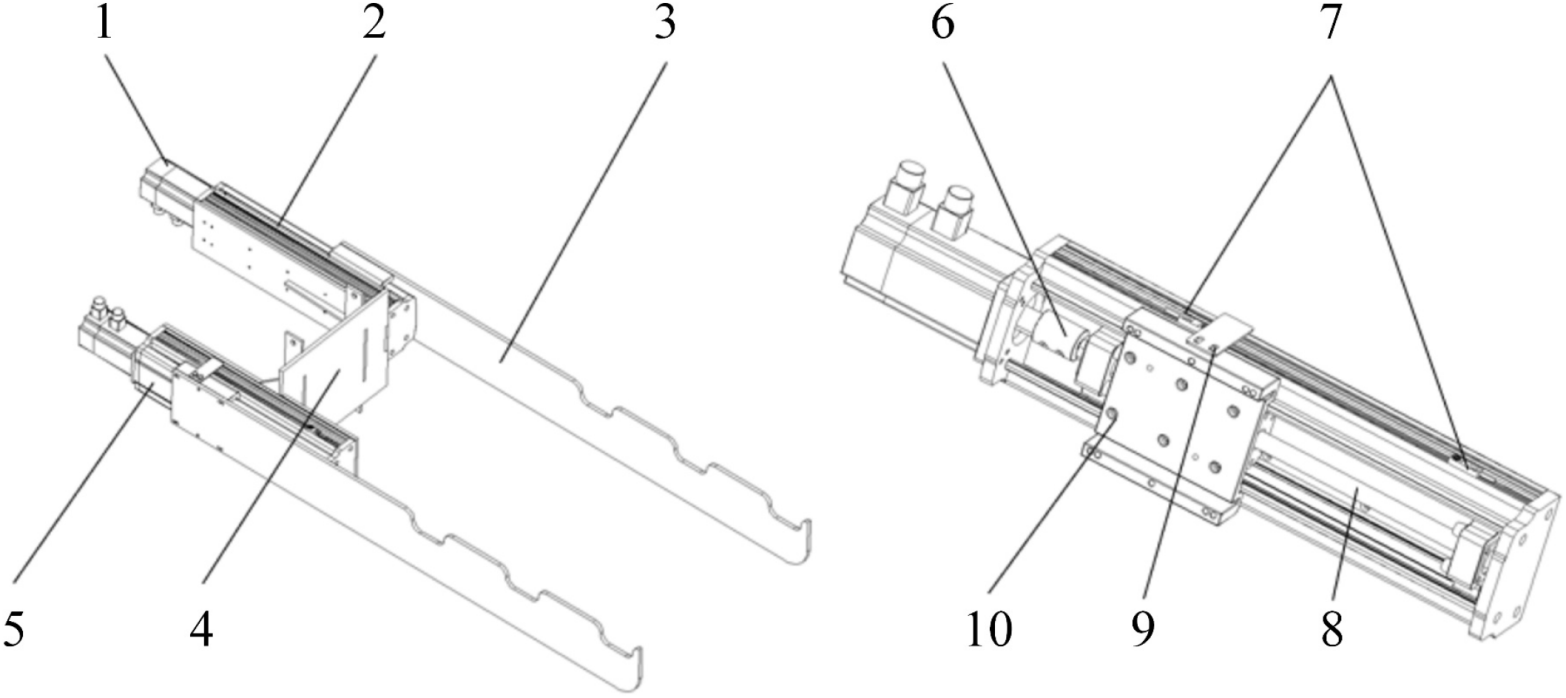
Structural schematic diagram of the tobacco-carrying device. Stepper motor 2. Linear module 3. Tobacco-carrying blade 4. Mounting plate of the tobacco-carrying device 5. Dustproof cover plate 6. Coupling 7. Proximity switch 8. Guide rail 9. Metal shield plate 10. Slider.

As one of the core structures of the tobacco stick transport and loading machine, the tobacco-carrying device is primarily designed to prevent interference between the tobacco sticks and the walls of the curing barn during transfer, ensuring the smooth execution of the hanging process. Based on the physical characteristics of tobacco sticks and the specific requirements of intensive curing barns, a reciprocating tobacco-carrying device was developed, as illustrated in **Figure 12**. The device mainly consists of a stepper motor, linear modules, tobacco-carrying blades, and a mounting plate. The mounting plate serves as the intermediate interface connecting the lifting mast to the linear modules; it is attached to the fifth-stage mast via two vertical plates, while two linear modules are secured to the plate with bolts. To achieve a lightweight design without compromising structural strength, the mounting plate is fabricated from aluminum alloy, significantly reducing the overall mass. The linear module is the core kinetic component of the device and comprises proximity switches, a guide rail, metal shield plates, and a slider. The module is bolted to the mounting plate, and the stepper motor is coupled to the guide rail via a coupling. A 140mm×140mm slider is mounted on the rail, equipped with a metal shield plate on one side. Two proximity switches are installed on the corresponding wall of the linear module for position detection and limit control. The tobacco-carrying blades are mounted on the slider, enabling horizontal reciprocating motion along the guide rail.

The two linear modules are arranged in a staggered configuration. Upon reaching the tobacco-collection point, the slider of the left module is positioned at the rear proximity switch, while the slider of the right module is at the front proximity switch. At this stage, the grooves on the two tobacco-carrying blades are perfectly aligned, allowing the tobacco stick to be placed horizontally.

Once the collection is complete, the left slider moves forward. When the proximity switch at the front of the left module detects the metal shield, the left stepper motor stops, indicating the blade has reached its designated position. Simultaneously, the right stepper motor rotates in the opposite direction, driving the right blade backward until the rear proximity switch detects its shield. This relative displacement between the two blades causes the tobacco stick to transition from a horizontal to a tilted orientation. To restore the horizontal state for the hanging operation, both stepper motors are restarted simultaneously in the reverse directions. The tilt angle of the tobacco stick plays an important role in maintaining transport stability and preventing unintended contact between the leaf tips and the curing barn walls during movement, thereby reducing the risk of mechanical damage to the leaves. It also affects the alignment accuracy during the hanging operation. Since the length of the tobacco stick is constant, the tilt angle varies according to the relative positions of the two blades. Therefore, a mathematical model relating the tilt angle to the relative blade positions is established to accurately describe the motion characteristics. Additionally, the relationship between the lateral distance and the blade positions is analyzed to prevent mechanical interference with the curing barn walls or other equipment, ensuring the safety and reliability of the transfer process.

**Figure 13:**
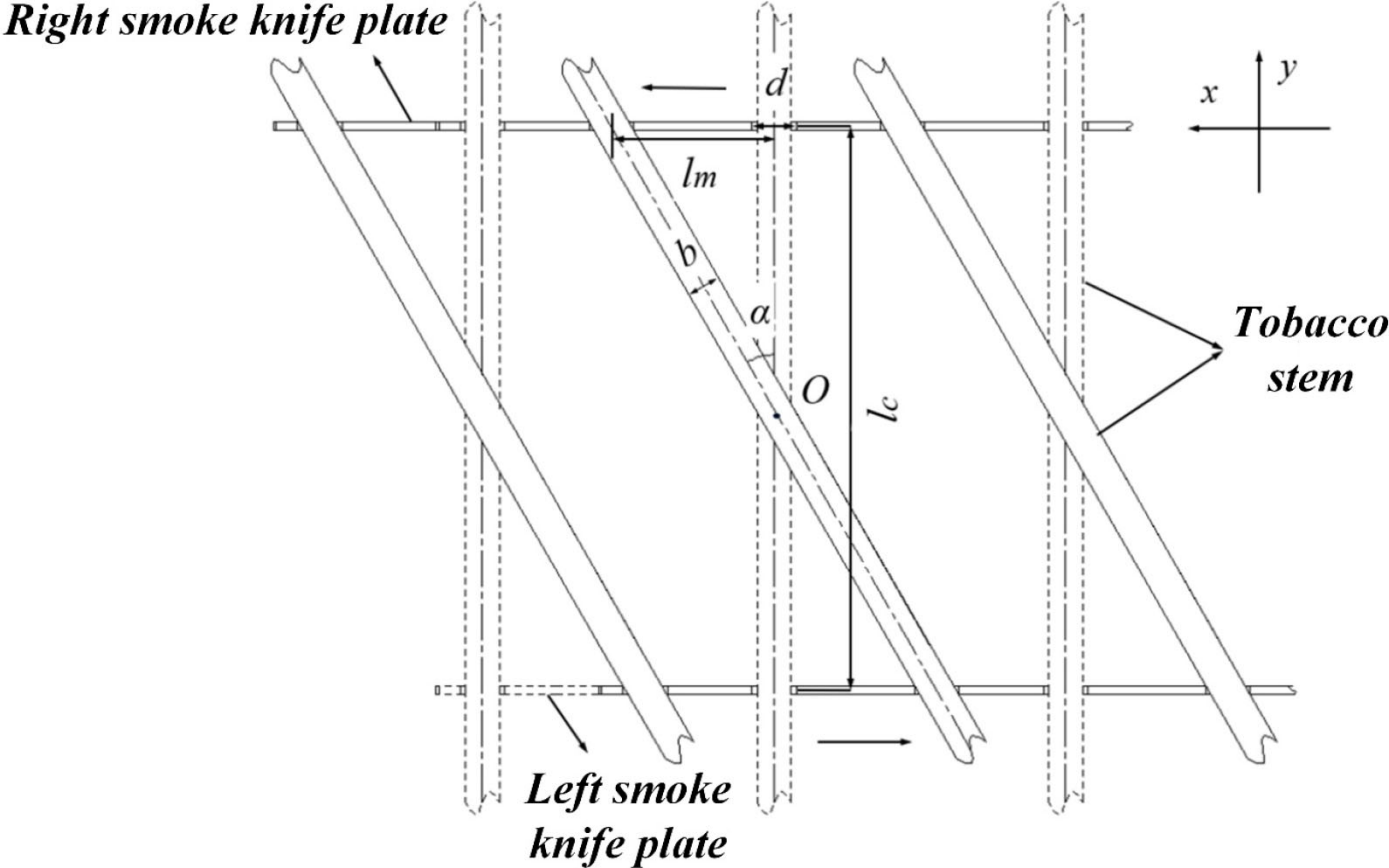
Schematic diagram of the motion of the tobacco-carrying device.

A top-down view of the tobacco stick and the carrying blades is shown in **Figure 13**. Taking the middle tobacco stick and the blades as an example for motion analysis: in the initial state, the grooves of the left and right blades are aligned, and the tobacco stick is perpendicular to the blades. As the tilting process commences, the right blade moves in the positive x-direction, while the left blade moves in the negative x-direction, causing the tobacco stick to rotate counter-clockwise around point O by an angle α. During this movement, the projected length of the tobacco stick in the y-direction is defined as $l_{d}$, which is closely correlated with the tilt angle α. A larger tilt angle results in a shorter projected length $l_{d}$, thereby increasing the clearance between the tobacco stick and the curing barn walls during transfer. This mechanism significantly reduces the risk of leaf damage caused by collisions with the walls. Furthermore, a specific mathematical relationship exists among the travel distance of the blades S, the spacing between the blades $l_{c}$, and the rotation angle α. For a given rotation angle, a smaller spacing between the blades requires a shorter travel distance for the blades. This allows for a more compact overall dimension of the carrying device, further enhancing the structural stability.

(13) $$\{\begin{array}{c} S=\frac{l_{c}}{2}\tan\alpha \\ \begin{array}{l} \alpha_{\min}=\arccos\frac{l_{b}}{l_{a}}\\ \alpha =\arccos\frac{{\text{l}}_{\text{d}}}{{\text{l}}_{\text{a}}} \end{array} \end{array}$$

Where,

S is the travel distance of the tobacco-carrying blades, *mm*;

$l_{a}$ is the length of the tobacco stick, *mm*;

$l_{b}$ is the width of a single-side loading chamber in the intensive curing barn, *mm*;

$l_{c}$ is the distance between the two blades, *mm*;

α is the rotation angle of the tobacco stick,°; $\alpha_{min}$ is the minimum rotation angle required to avoid interference between the tobacco stick and the curing barn walls,°.

**Figure 14:**
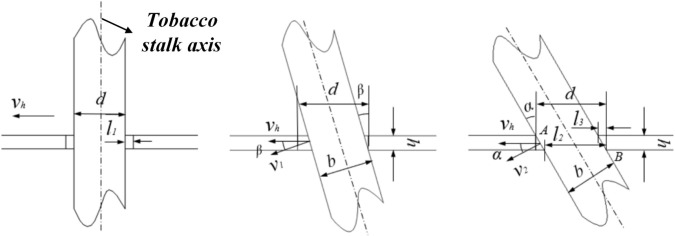
Schematic diagram of the tilting motion process of the tobacco stick.

Based on the structural characteristics of intensive curing barns and the methods used for tobacco loading, the length of the tobacco stick $l_{a}$ ranges from 1360 to 1400 mm, while the width of a single-side loading chamber $l_{b}$ is 1,350 mm. By substituting these known parameters into Equation 13, the minimum rotation angle $\alpha_{min}$ is calculated to be 15.35°. Therefore, the rotation angle of the tobacco stick must exceed 15.35° to prevent collisions with the walls when the machine enters the curing barn. However, considering that the interior of most curing barns consists of unrefined concrete pavement with potential pits and bumps, and accounting for possible tracking deviations of the machine along the magnetic strip, a sufficient safety clearance must be maintained between the tilted tobacco stick, the walls, and the hanging racks. Through an analysis of the barn’s structural parameters, it is stipulated that the distance between the ends of the tilted stick and the walls should be at least 80 mm. Under this condition, the projected length of the tobacco stick in the *y*-direction $l_{d}$ is 1,190 mm. Substituting this value into Equation 13 yields a required rotation angle α of 31.86° and a corresponding travel distance for the tobacco-carrying blades S of 155.99 mm. The motion process of the tobacco stick during the tilting phase is further analyzed. In the initial state, the tobacco stick is positioned perpendicularly to the tobacco-carrying blades, with a clearance distance ${\text{l}}_{1}$ maintained between the stick and the two side walls of the blade groove. Upon the commencement of the tilting motion, the carrying blade moves along the positive *x*-direction at a velocity $v_{h}$. Under the influence of frictional force f, the tobacco stick shifts in the same direction. Once it makes contact with the right wall of the groove, the stick begins to perform a rotational motion around point O at a linear $v_{h}$ velocity, driven by the support force exerted by the right wall. As the tilting progresses, the rotation velocity $v_{1}$ varies according to the change in the rotation angle. A larger rotation angle results in a corresponding decrease in the rotation velocity $v_{1}$, until the carrying blade reaches its designated position and the tilting is completed. At this final stage, the tobacco stick intersects the two side walls of the groove at points A and B, achieving the final rotation angle α. Based on the schematic diagram of the tilting motion process of the tobacco stick shown in **Figure 14**, Equation 14 can be obtained.

(14) $$\{\begin{array}{c} \begin{array}{c} l_{1}=\frac{d-b}{2}\\ v_{1}=v_{h}\cdot \cos\beta \\ l_{2}=\frac{b}{\sin\left(\frac{\pi }{2}-\alpha \right)}\\ l_{3}=h\cdot \tan\alpha \end{array}\\ d=l_{2}+l_{3} \end{array}$$

Where,

$l_{1}$ is the clearance distance between the tobacco stick and the groove wall at the initial position, *mm*;

d is the width of the groove on the tobacco-carrying blade, *mm*;

b is the diameter of the tobacco stick, *mm*;

$v_{1}$ is the rotational velocity of the tobacco stick, *m/s*;

h is the thickness of the tobacco-carrying blade, *mm*.

Given that the rotation angle α is 32° when the tobacco stick is tilted into position, the stick diameter *b* ranges from 27 to 33 mm, and the blade thickness *h* is 10 mm, it can be calculated that $l_{2}$ ranges from 34.17 to 41.76 mm, $l_{3}$=5.49mm, and the range ford is 39.66 to 47.25 mm. Considering that some tobacco sticks exhibit natural curvature, a safety margin was incorporated into the design. Consequently, the groove width of the tobacco-carrying blade was set to 50 mm to prevent the sticks from snapping due to torsional stress during the tilting process.

## 3 Finite element analysis of key components

### 3.1 Static analysis of the power chassis and mast assembly

**Figure 15:**
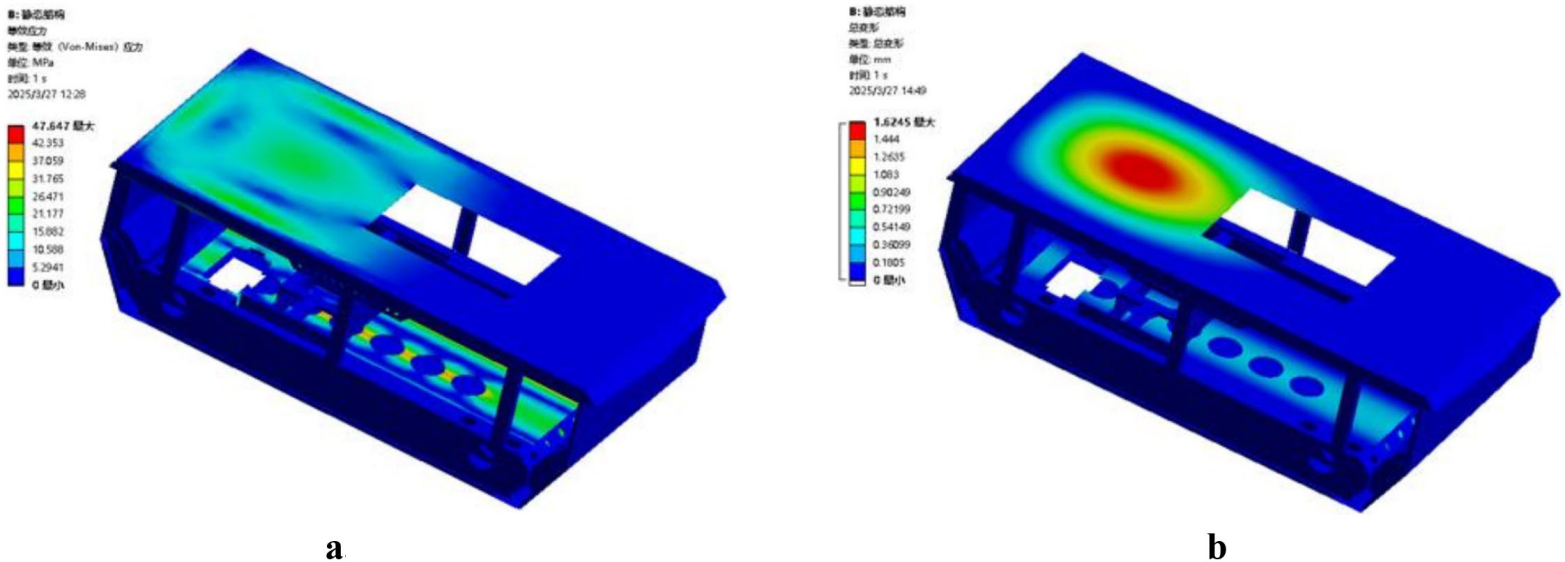
Static analysis of the power chassis frame. **(a)** Equivalent stress contour. **(b)** Total deformation contour.

In this study, the static analysis of the power chassis frame and the mast assembly was conducted using ANSYS Workbench. During the construction of the geometric model, a simplification principle was applied, retaining only the primary load-bearing components: the chassis frame and the telescopic mast assembly. The crossbeams and longitudinal beams of the frame are welded from Q235 structural steel. To enhance structural integrity, the sidewalls of the frame are reinforced with ten U-shaped steel stiffeners, also made of Q235. The mast assembly is fabricated from 6005 aluminum alloy, which offers a high strength-to-weight ratio while satisfying rigorous strength requirements. A structured meshing approach using hexahedral elements was employed for both the frame and the mast assembly. In critical load-bearing regions-such as the junctions of the longitudinal beams and the contact surfaces of the mast guide rails-a fine mesh of 5 mm was applied. Conversely, a relatively coarse mesh of 10 mm was used for non-load-bearing areas, such as the top plate of the frame and non-contact segments of the masts. The mesh quality metrics for the frame showed a Skewness of 0.19 and an Element Quality of 0.79, resulting in a total of 238,695 nodes and 11,864 elements. For the mast assembly, the Skewness and Element Quality were 0.17 and 0.83, respectively, with a total of 284,020 nodes and 140,923 elements. After setting the boundary conditions and applying the loads to the power chassis frame and the mast assembly, the resulting stress-strain distribution characteristics are illustrated in **Figure 15**. Analysis of the equivalent stress contour reveals that the maximum equivalent stress of the frame is 47.647 MPa, with the stress concentration primarily localized at the load-bearing center of the frame’s base. The frame is constructed from Q235 structural steel, which has a yield strength of 235 MPa. Since the maximum stress experienced by the frame is significantly lower than the material’s yield limit, it is verified that the chassis structure fully satisfies the load-bearing requirements of the transport and loading machine under various operating conditions. As shown in the strain contour in **Figure 15(b)**, the deformation is mainly concentrated on the cover plate that supports the weight of the control box. The maximum deformation is 1.625 mm, which is considered a minor deformation and has a negligible impact on the overall structural integrity of the frame. However, to enhance the engineering safety factor, a 5 mm thick Q235 reinforcing steel plate was added to the mounting surface of the control box cover in the final design.

**Figure 16:**
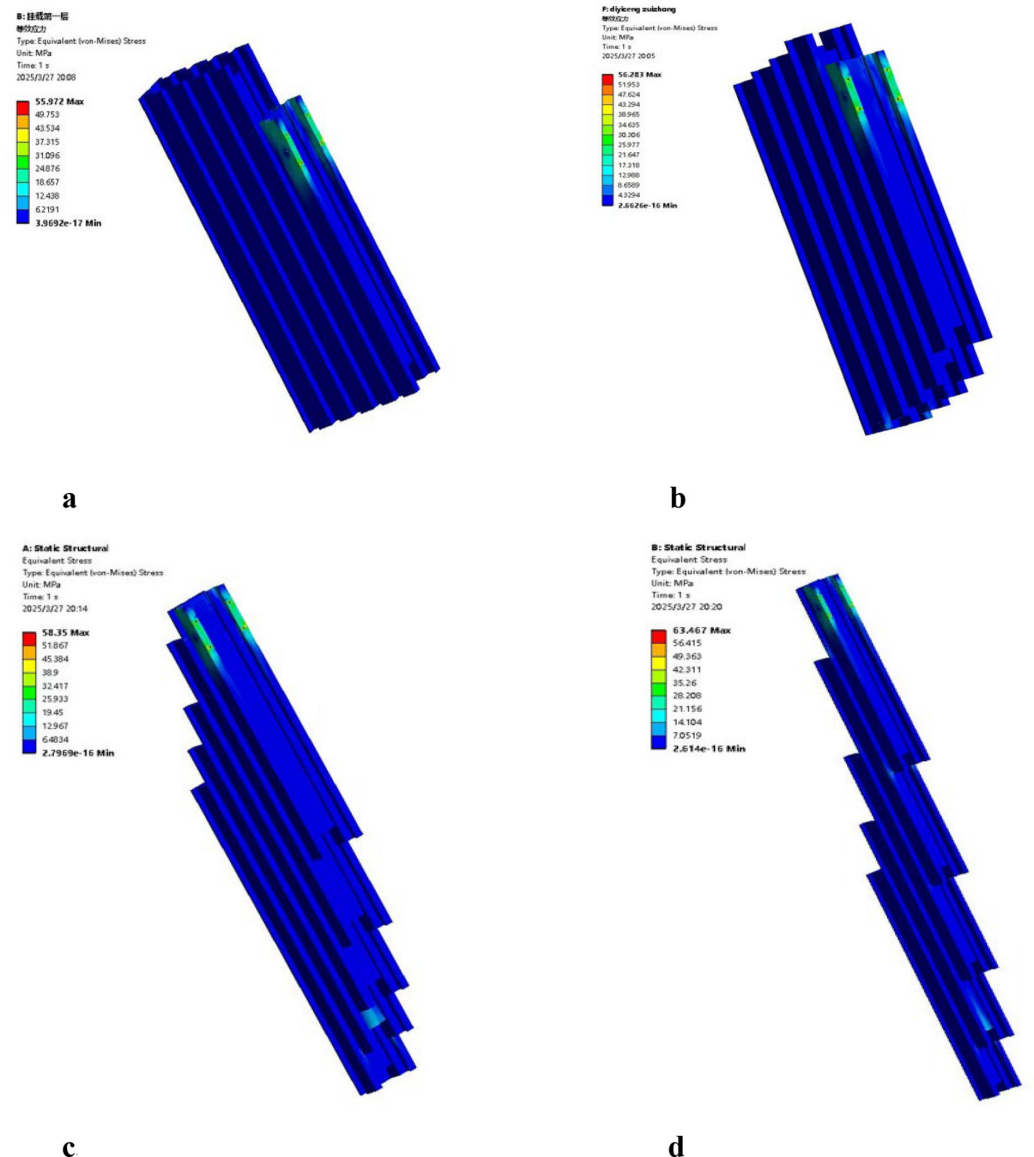
Equivalent stress contours of the mast assembly under different operational conditions. **(a)** operational condition 1. **(b)** operational condition 2. **(c)** operational condition 3. **(d)** operational condition 4.

During the analysis of the mast assembly, four specific operational conditions (OC) were defined based on the lifting state and loading positions while fully loaded with tobacco sticks: OC 1: Lifting state at the initial (lowest) position; OC 2: Lifting state while hanging at the bottom tier; OC 3: Lifting state while hanging at the middle tier; OC 4: Lifting state while hanging at the top tier. The equivalent stress contours for these four conditions are illustrated in **Figure 16**. The simulation results indicate that the maximum stress values for OC 1, OC 2, OC 3, and OC 4 are 55.97 MPa, 56.28 MPa, 58.35 MPa, and 63.46 MPa, respectively. In all cases, the maximum stress is concentrated at the bolted joints between the tobacco-carrying device mounting plate and the fifth-stage mast. The mast assembly is fabricated from 6005 aluminum alloy with a yield strength of 215 MPa. Comparing the yield strength to the maximum calculated stress, the resulting ratios all exceed the design safety factor of 3.0, which is within the recommended safety factor range for load-bearing mechanical components under static conditions (Budynas and Nisbett, 2015). Therefore, the structural design of the mast assembly satisfies the strength requirements under all operational conditions and demonstrates sufficient structural integrity.

**Figure 17:**
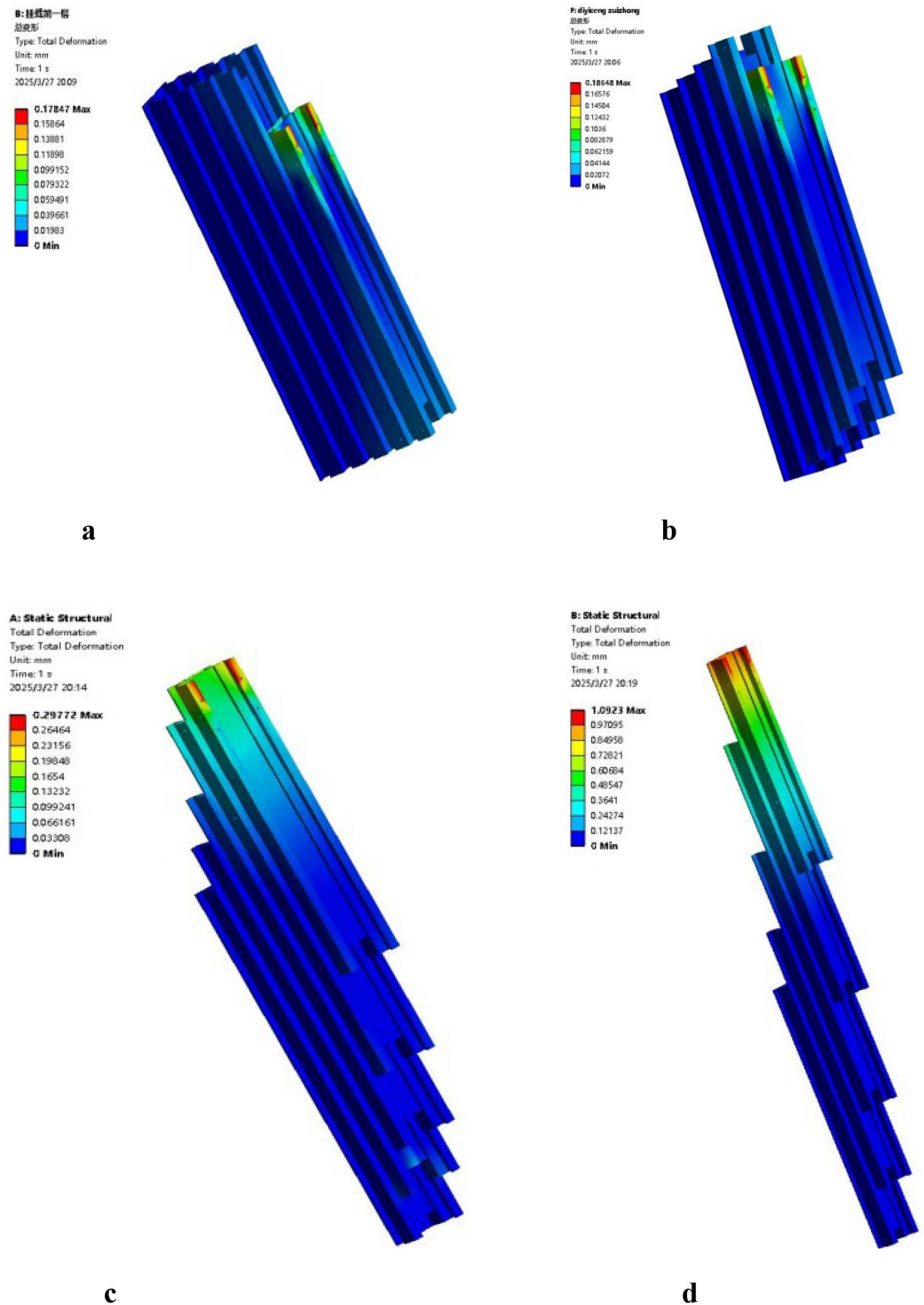
Total deformation contours of the mast assembly under different operational conditions. **(a)** operational condition 1. **(b)** operational condition 1. **(c)** operational condition 3. **(d)** operational condition 4.

The total deformation contours of the mast assembly under the four operational conditions are illustrated in **Figure 17**. The maximum deformation values for OC 1, OC 2, OC 3, and OC 4 are 0.18 mm, 0.19 mm, 0.29 mm, and 1.10 mm, respectively. These results indicate that as the lifting height increases, the deformation of the fifth and fourth-stage masts progressively grows. Notably, the maximum deformation in OC 4 accounts for only 0.031% of the maximum lifting height of the mast assembly. This value is significantly lower than the standard regulatory requirement, which stipulates that the maximum deflection shall not exceed 0.5% of the maximum lifting height, in accordance with established structural deformation control principles in mechanical design (Budynas and Nisbett, 2015). Therefore, the deformation characteristics of the mast assembly under all operational conditions satisfy the design requirements.

### 3.2 Modal analysis of the mast assembly

**Figure 18:**
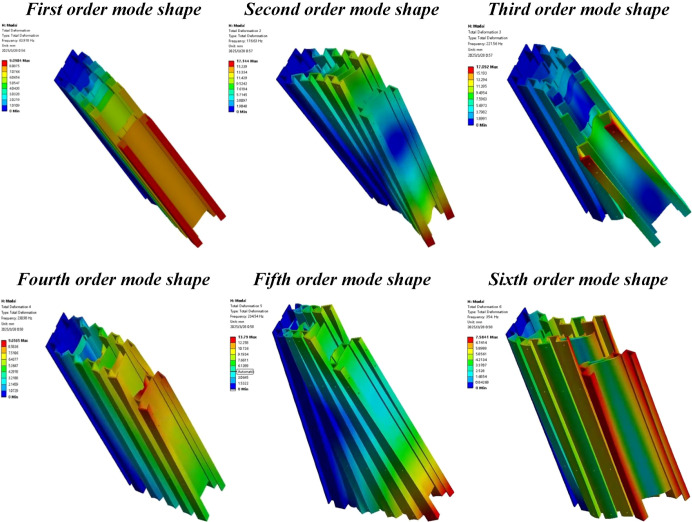
Mode shape diagrams of the first six orders for the mast assembly.

The mast assembly is integrated with the power chassis through the mounting plate of the lifting device. In operating environments such as intensive curing barns, the tobacco transport and loading machine may encounter excitation forces when crossing door sills or traveling on unpaved surfaces. These excitation forces are transmitted to the mast assembly via the tires. Furthermore, vibrations are easily generated during the operation of the servo motors and the lead screw elevators. When the external vibration frequency applied to the mast assembly matches its natural frequency, a resonance effect occurs. This can lead to structural instability and intensified deformation, severely compromising the operational performance of the equipment (Zhang, 2023). The mast assembly model in its initial position was imported into the ANSYS Model module. The material was defined as 6005 aluminum alloy, and the solution was executed following mesh generation and the configuration of boundary conditions. Since most external excitation frequencies are low-order and are more likely to trigger low-order resonance, this study focuses on analyzing only the first six mode shapes of the mast assembly. The results of the first six modal orders are summarized in **Table 4**, and the corresponding mode shape diagrams are illustrated in **Figure 18**.

**Table 4:** Modal analysis results of the mast assembly.

Order	Natural frequency/(Hz)	Maximum displacement/(mm)
1	63.92	9.10
2	176.63	17.14
3	221.56	17.09
4	230.98	9.66
5	234.54	13.78
6	354.00	7.58

The results indicate that the natural frequencies and corresponding mode shapes of the mast assembly are as follows: The 1st order natural frequency is 63.92 Hz, and the mode shape exhibits bending deformation along the positive x-axis. The 2nd and 3rd order natural frequencies are 176.63 Hz and 221.56 Hz, respectively, with mode shapes characterized by torsional oscillation along the x-direction. The 4th order natural frequency is 230.98 Hz, manifesting as tensile deformation in the y-direction. The 5th and 6th order natural frequencies are 234.54 Hz and 354.00 Hz, respectively, with mode shapes showing torsional oscillation along the y-direction. During the operation of the transport and loading machine, the external excitations acting on the mast assembly primarily originate from the vibrations of the power chassis caused by ground irregularities and the high-speed rotation of the servo motors during lifting operations. According to the design specifications, the maximum speed of the servo motor is 300 rpm, which corresponds to a frequency of 5 Hz. Furthermore, ground-induced vibrations are typically low-order frequencies, generally falling within the range of 1–5 Hz. Consequently, the frequency range of external excitations does not overlap with any of the natural frequencies of the mast assembly, ensuring that resonance will not occur.

### 3.3 Fatigue analysis of the tobacco-carrying knife plate

**Figure 19:**
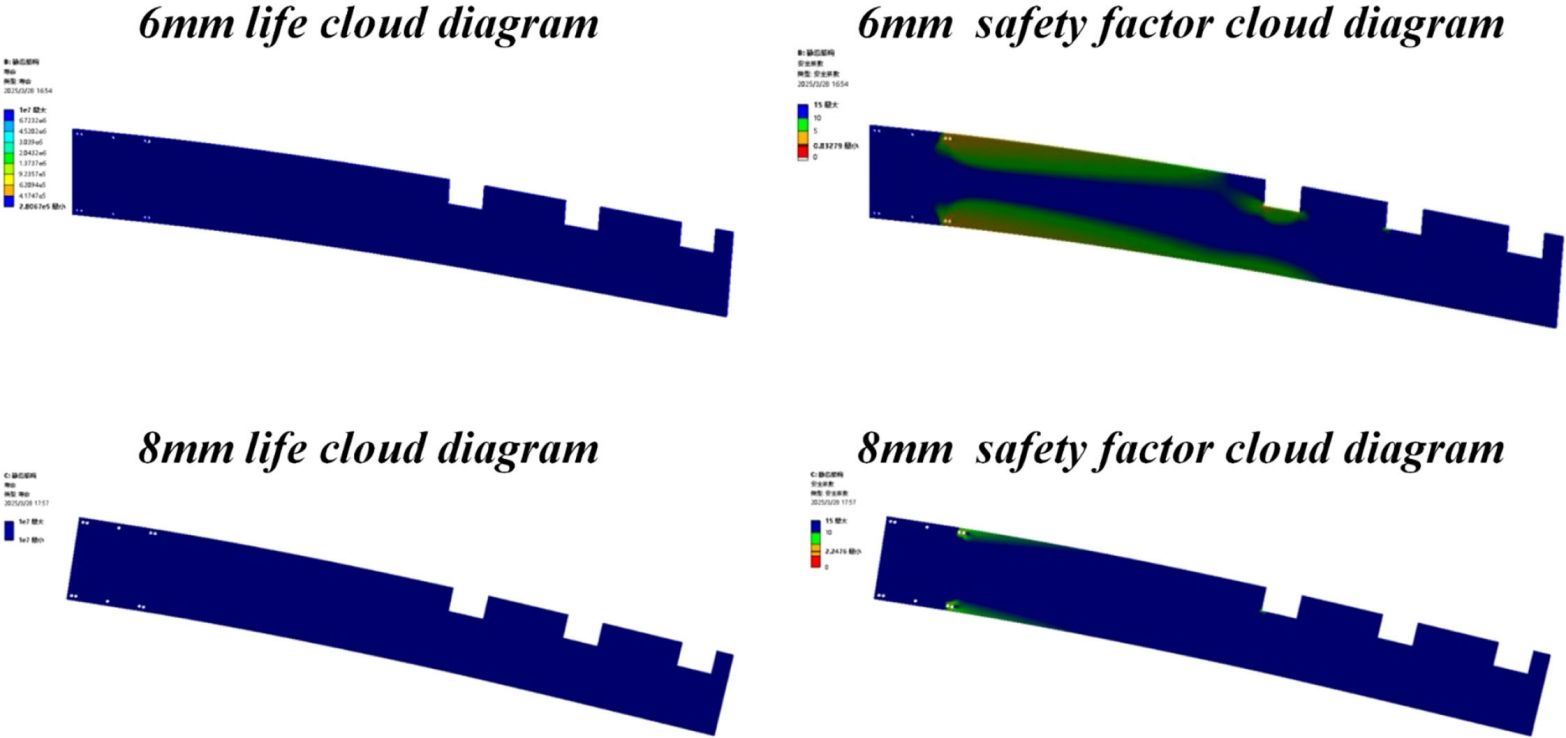
Fatigue life and safety factor contours of the tobacco-carrying blade.

As a critical load-bearing component of the transport and loading machine, the tobacco-carrying knife plate is subjected to frequent alternating loads during loading and unloading operations. To minimize mechanical damage to the tobacco leaves during transit, the knife plate thickness should be kept as thin as possible. However, such a design makes the knife plate susceptible to fatigue fracture under alternating stress, posing a threat to the overall safety of the machine. Therefore, the *S-N* nominal stress method was employed to conduct a fatigue analysis of the tobacco-carrying knife plate. Based on the results from ANSYS Workbench (as shown in **Figure 18**), knife plates of different thicknesses exhibited significant differences in fatigue performance. According to the characteristics of the material’s *S-N* curve (Liu et al., 2019), a threshold of 106 cycles was defined as the fatigue life limit, beyond which the component is considered to be in an infinite life state. For the 6 mm thick knife plate, the results indicate that while most regions meet the 106 cycles life requirement and theoretically satisfy normal operational demands, the safety factor distribution contour (as shown in **Figure 19**) reveals a local minimum safety factor of only 0.84. This value falls below the engineering safety threshold of 1.0, indicating a potential risk of fatigue failure during actual operation. To enhance structural reliability, a comparative analysis was performed by increasing the knife plate thickness to 8 mm. The results demonstrate that the improved design not only satisfies the 106 cycles life requirement across all regions but also increases the minimum safety factor to 2.24, which is significantly higher than the allowable value. This optimization effectively eliminates potential fatigue failure risks and complies with fatigue life design specifications.

### 3.4 Experimental verification

**Figure 20:**
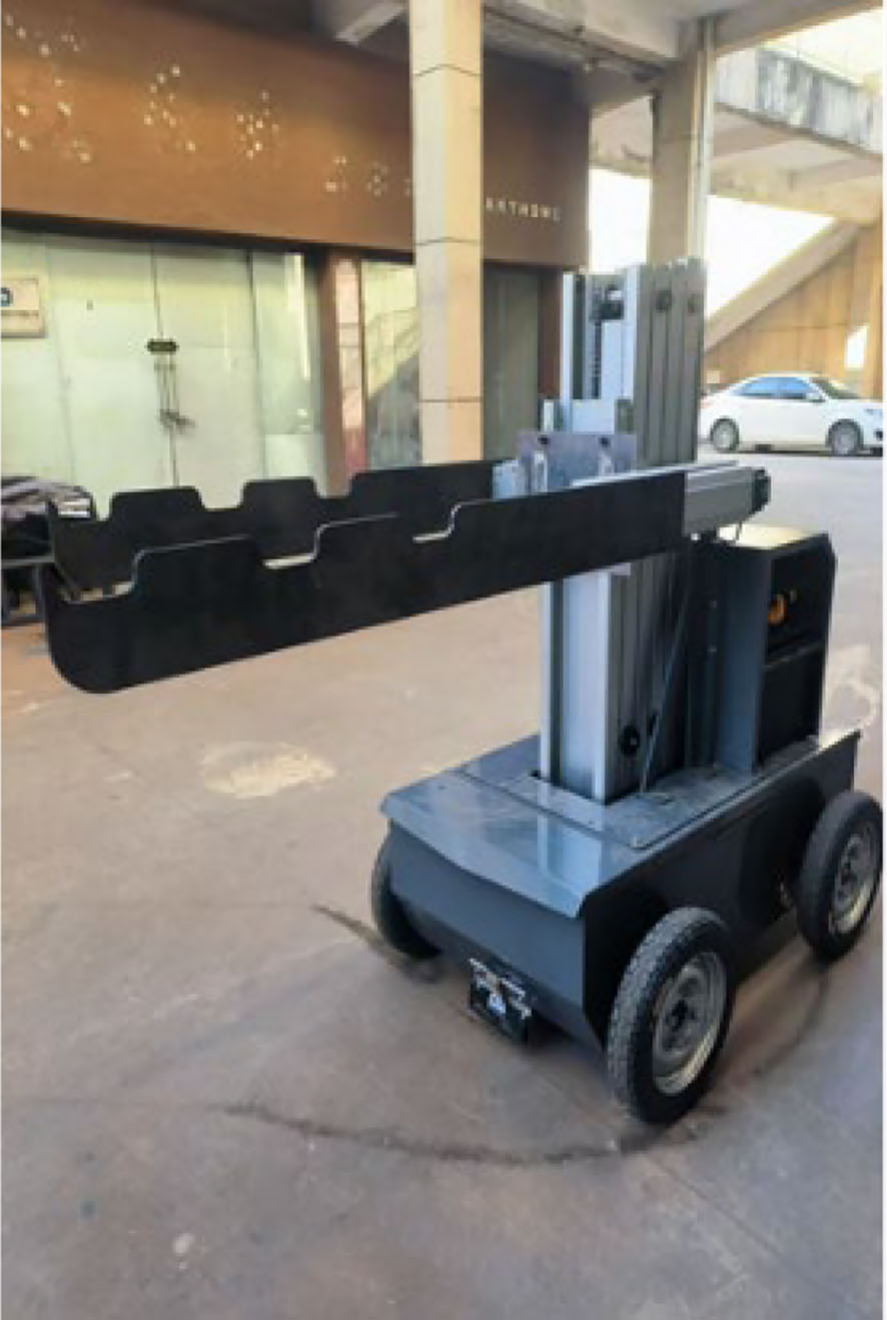
Prototype of the tobacco transport and loading machine.

Based on the structural design of the tobacco transport and loading machine, the component fabrication and system assembly were commissioned to Taian Shuobao Intelligent Technology Co., Ltd. **Figure 20** shows the prototype of the tobacco transport and loading machine. The field trials were subsequently conducted at the intensive curing barns within the Fangyuan Tobacco Scientific Research Base in Guiyang County, Chenzhou City, Hunan Province. The experimental curing barn is a horizontal structure built according to the Technical Specifications for Intensive Curing Barns, with dimensions of 8.0 m × 2.7 m × 3.5 m (L × W × H). The tobacco sticks used for the trial were wooden or bamboo poles with lengths ranging from 130 mm to 135 mm. Since the tobacco leaves had not yet reached maturity during the testing period, celery was employed as the experimental material due to its physical characteristics being similar to those of fresh tobacco leaves.

To evaluate the operational efficiency and loading performance of the tobacco transport and loading machine, a comparative experiment was designed between manual loading and machine-assisted loading. Since the curing barn is divided into three tiers (top, middle, and bottom), loading tests were conducted for each tier separately to verify the machine’s performance at different heights. Each group of experiments was repeated 10 times (resulting in a total of 30 tobacco sticks being loaded). During the trials, relevant data for both manual and machine-assisted loading were recorded to analyze the differences in efficiency and performance. The detailed experimental scheme is presented in **Table 5**.

**Table 5:** Experimental scheme for tobacco loading.

Loading method	Sticks per loading	Labor required (Persons)	Trials per group
Manual Loading	1	2	3
Tobacco Transport and Loading Machine	3	1	1

The loading success rate and the loading efficiency were selected as the primary evaluation indices for the experiments. Loading Success Rate (*S*): The proportion of tobacco sticks successfully placed into the designated positions by the transport and loading machine. Loading Efficiency (*V*): The number of tobacco sticks successfully loaded per person per unit of time. The calculation methods are expressed in Equations 15 and 16:

(15) $$\text{S}=\frac{\text{N}}{M}\times 100\%$$

(16) $$\text{v}=\frac{\text{N}}{\text{m}\sum_{\text{i}=1}^{10}{\text{T}}_{\text{i}}}$$

Where:

N —Number of successfully loaded tobacco sticks (*sticks*); M—Total number of tobacco sticks attempted for loading (*sticks*);

m—Number of operators involved in the loading process;

$T_{I}$—Time consumed for the i-th group of loading operations (*min*).

To further quantify the relative operational performance between manual loading and machine-assisted loading, an Efficiency Ratio (R) was introduced and defined as follows:

(17) $$R=\frac{V_{machine}}{V_{manual}}$$

Where $V_{machine}$ and $V_{manual}$represent the loading efficiency under machine-assisted and manual loading conditions, respectively.

As shown in **Table 6**, the tobacco transport and loading machine demonstrates a clear advantage over manual loading in terms of operational efficiency. Regarding the loading success rate, the manual groups maintained a 100% success rate across all tiers, while the machine groups achieved success rates ranging from 90% to 96.7%. The slightly lower success rate of the machine was attributed to human error during the initial placement of the tobacco sticks onto the carrier. If the center of the stick was not precisely aligned with the centerline of the loading device, the stick would become unbalanced during the transition from an inclined state to a horizontal state. This misalignment caused one end of the stick to be successfully seated on the curing rack while the other end failed to catch. Despite this, the data analysis confirms that the designed machine can effectively execute transport and loading tasks, corresponding to efficiency ratios ranging from 2.89 to 4.60, as calculated using Equation 17.

**Table 6:** Comparison of operational efficiency between different loading schemes.

Category	Times	Successfully loaded sticks (sticks)	Success rate (%)	Loading efficiency (sticks/person·min)	Efficiency ratio
Manual	1	30	100	3	–
	2	30	100	2.7	–
	3	30	100	1.5	–
Machine	1	28	93.3	8.9	2.97
	2	29	96.7	7.8	2.89
	3	27	90	6.9	4.60

Although manual loading achieved a 100% success rate in the experimental trials, the machine-assisted loading exhibited success rates ranging from 90% to 96.7%. The observed failures were primarily attributed to slight positional deviations during the initial placement of tobacco sticks onto the carrying mechanism. Specifically, when the longitudinal center of the tobacco stick was not precisely aligned with the centerline of the loading device, imbalance occurred during the transition from the inclined state to the horizontal hanging state. This misalignment could cause one end of the stick to engage properly with the curing rack while the opposite end failed to seat securely. To address this issue, future improvements will focus on enhancing the positioning accuracy and tolerance adaptability of the carrying mechanism. Potential optimization measures include the integration of auxiliary alignment guides, adaptive clamping structures, or sensor-assisted feedback mechanisms to automatically detect and correct misalignment prior to the hanging process. These refinements are expected to enhance loading reliability without compromising the efficiency performance demonstrated by the current prototype.

## 4 Conclusion

1. Based on the structural characteristics of intensive curing barns and conventional tobacco loading requirements, a modular design philosophy is adopted to propose a comprehensive design for the tobacco transport and loading machine. This design integrates the power chassis, lifting device, tobacco-carrying device, and control box. The operational stability of the machine under extreme working conditions is analyzed. The results indicate that the machine’s reliability and stability satisfy all design requirements, even under high-load and high-altitude operations.
2. A five-stage telescopic mast lifting device and a reciprocating tobacco-carrying device are innovatively designed, enabling precise lifting within a range of 1.3–3.6 m and tobacco stick inclination adjustments from 0° to 32°. This design effectively prevents mechanical interference between the machine and the curing barn walls. Finite element analysis demonstrates that the strength of the primary load-bearing structures, such as the telescopic masts, meets safety standards. The first six natural frequencies of the mast assembly range from 63.92 to 354.00 Hz, ensuring that resonance will not occur. Furthermore, the safety factor of the tobacco-carrying blades is significantly higher than the allowable value, complying with fatigue life design specifications.
3. Field trial results conducted in tobacco pole production areas show that the loading success rate of the machine exceeds 90%. Compared to traditional manual operations, the operational efficiency has been improved by approximately 3 to 5 times. The tobacco transport and loading machine can effectively execute stick transport and hanging tasks, significantly enhancing overall productivity.

## Data Availability

The original contributions presented in the study are included in the article/supplementary material. Further inquiries can be directed to the corresponding author.
